# Evolution of Conserved Noncoding Sequences in *Arabidopsis thaliana*

**DOI:** 10.1093/molbev/msab042

**Published:** 2021-02-10

**Authors:** Alan E. Yocca, Zefu Lu, Robert J. Schmitz, Michael Freeling, Patrick P. Edger

**Affiliations:** 1 Department of Plant Biology, Michigan State University, East Lansing, MI, USA; 2 Department of Horticulture, Michigan State University, East Lansing, MI, USA; 3 Department of Genetics, University of Georgia, Athens, GA, USA; 4 Department of Plant and Microbial Biology, University of California, Berkeley, CA, USA; 5 Ecology, Evolutionary Biology and Behavior, Michigan State University, East Lansing, MI, USA

**Keywords:** Molecular evolution, conserved noncoding sequence, intraspecific genomics

## Abstract

Recent pangenome studies have revealed a large fraction of the gene content within a species exhibits presence–absence variation (PAV). However, coding regions alone provide an incomplete assessment of functional genomic sequence variation at the species level. Little to no attention has been paid to noncoding regulatory regions in pangenome studies, though these sequences directly modulate gene expression and phenotype. To uncover regulatory genetic variation, we generated chromosome-scale genome assemblies for thirty *Arabidopsis thaliana* accessions from multiple distinct habitats and characterized species level variation in Conserved Noncoding Sequences (CNS). Our analyses uncovered not only PAV and positional variation (PosV) but that diversity in CNS is nonrandom, with variants shared across different accessions. Using evolutionary analyses and chromatin accessibility data, we provide further evidence supporting roles for conserved and variable CNS in gene regulation. Additionally, our data suggests that transposable elements contribute to CNS variation. Characterizing species-level diversity in all functional genomic sequences may later uncover previously unknown mechanistic links between genotype and phenotype.

## Introduction

**Fig. 1.**
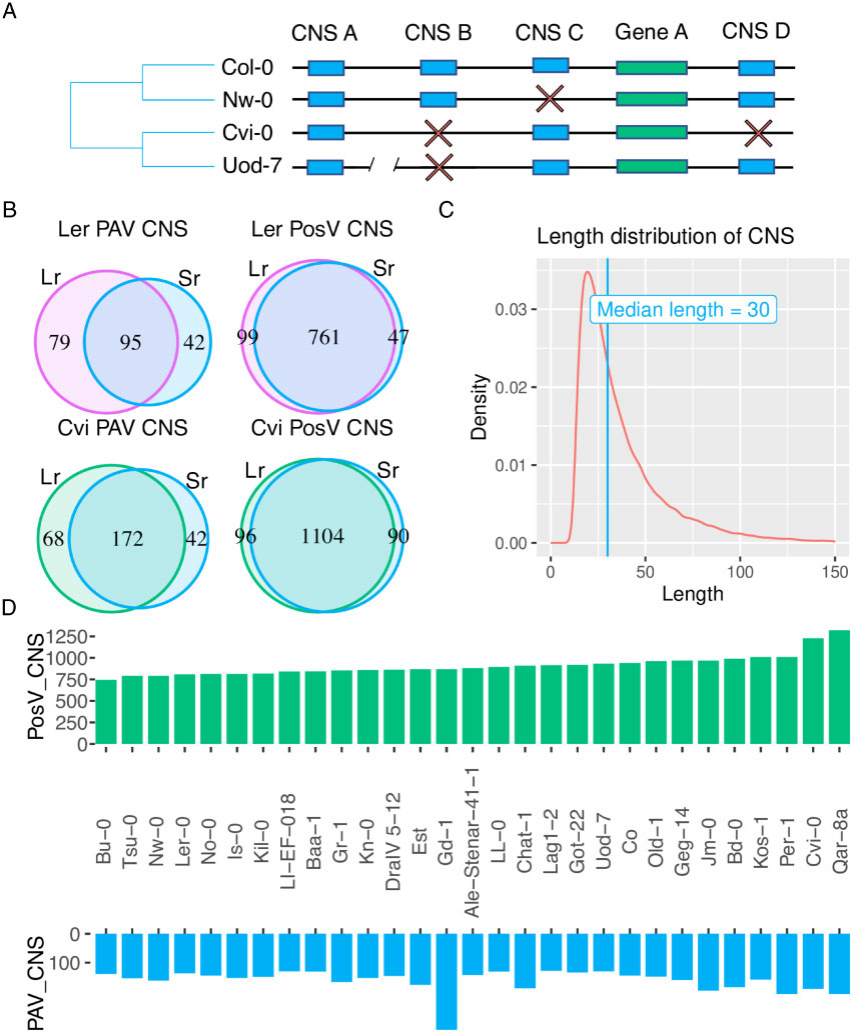
(*A*) A model depicting intraspecific variation in CNS content. Blue blocks represent conserved non-coding sequences (CNS) and green blocks represent genes. CNS may occur upstream or downstream genes. Red “X” characters depict the failure to identify a CNS in an A. thaliana accession in the position at which we find the CNS in the reference accession Col-0. CNS A in accession Uod-7 is found at a location other than where it is found in Col-0 as demarcated by the genomic position break. Therefore, in accession Uod-7, CNS A displays PosV. (*B*) Venn diagrams comparing the CNS variation observed in identical accessions between publically available long-read (Lr) genome assemblies and those generated with short-reads (Sr) in this study. (*C*) The length distribution in base-pairs of the 62,916 CNS studied here showing a minimum sequence length of fifteen base-pairs, and a median of thirty base-pairs. (*D*) A mirrored bar chart showing the number of PosV (top) and PAV (bottom) CNS identified in each accession in this study. Accessions are sorted by the number of PosV CNS.

Conserved noncoding DNA remains a highly understudied class of functional genomic features compared to protein-coding genes. Previous comparative genomic analyses in plants have identified stretches, generally 15–150 base pairs (bp) long (fig. 1*C*), of noncoding regions with identical (or near identical) sequence across distantly related species (Haudry et al. 2013; Burgess and Freeling 2014; Van de Velde et al. 2016). These sequences, commonly referred to as Conserved Noncoding Sequences (CNS), are regions in the genome displaying much higher similarity across different taxa than expected by chance. Background mutation and genetic drift purges nonfunctional sequences over long evolutionary distances. Therefore, sequence conservation above expectation implies purifying selection actively conserves these CNS. Indeed, Williamson et al. (2014) discovered elevated signatures of purifying selection in CNS regions compared to other classes of noncoding DNA in *Capsella grandiflora* (Brassicaceae). Previous studies demonstrated that CNS contain transcription factor binding sites (TFBSs) (Burgess and Freeling 2014; Van de Velde et al. 2014; Xie et al. 2018).

TFBSs are typically 6–12 basepair (bp) long (Kulkarni et al. 2018). CNS can exceed this length, as they are thought to consist of arrays of TFBS capable of recruiting independent or cooperative transcriptional protein complexes. The length of CNS enables high confidence identification of orthologous *cis*-regulatory elements in other genomes. Querying genomes for TFBS alone results in a high false positive rate, as there are >30,000 expected occurrences of a given six bp sequence expected by chance even in the relatively small (∼135 Mb) *Arabidopsis thaliana* (Brassicaceae)genome. In contrast, there is less than one expected random occurrence of the shortest CNS (15 bp). TFBS colocalize with accessible chromatin in mammalian genomes, as do CNS as demonstrated previously in plants (Zhang et al. 2012; Van de Velde et al. 2016; Lu et al. 2017; Lai et al. 2017; Zhao et al. 2018; Lu et al. 2019; Ricci et al. 2019).

Genes experience a broad spectrum of selective forces potentially resulting in strong conservation (i.e., resisting deletion) (Birchler and Veitia 2012) or active removal from certain genomes (Sharma et al. 2018). Certain gene families are known to exhibit high birth–death dynamics, whereas other gene families are relatively stable in size (Edger and Pires 2009; Freeling 2009). Thus, some genes are present in all eukaryotes, whereas others may be lineage specific (Maere et al. 2005). Equivalently, a subset of CNS identified across Brassicaceae (Haudry et al. 2013) are identifiable across all surveyed angiosperms including *Amborella trichopoda* (Amborellaceae) (Burgess and Freeling 2014) whereas others are uniquely shared by only a subset of Brassicaceae.

Previous pangenome studies aimed to capture presence–absence variation (PAV) in transcribed regions to characterize the core and dispensable gene content (Golicz et al. 2016; Gordon et al. 2017; Montenegro et al. 2017; Hübner et al. 2019). These studies consistently find core genes (those present across most individuals within a species) are enriched in essential cellular processes, whereas dispensable genes often display higher mutation rates and are biased towards adaptive processes (e.g., response to the environment). We hypothesize dispensable CNS follow patterns observed for dispensable coding regions such as representing a pool of sequences contributing to adaptive processes and potentially important agronomic traits.

Though tens of thousands of CNS have already been identified in plant genomes, these comparisons are often performed between single representatives of select distantly related species. To our knowledge, the variation in CNS content across the genome of multiple individuals within a single species has never been addressed in plants. Here, we assembled chromosome-scale genomes for thirty *A. thaliana* accessions and leveraged one of the largest annotated CNS datasets (Haudry et al. 2013), to investigate the levels and patterns of intraspecific variation of CNS and the impact of this variation on gene expression in *A. thaliana*.

## Results

### What Proportion of CNS Vary within a Species?

CNS are typically identified through whole genome comparisons of single representative genomes of different species spanning various phylogenetic distances. Therefore, the variation of these sequences at the species level remains poorly understood, especially in plants. We investigated two main types of variation in CNS structure across multiple *A. thaliana* accessions: PAV and positional variation (PosV). We define PAV CNS as those present in the reference accession (Col-0), but absent in at least one other accession. PosV CNS are those which exist in a different locus in an accession relative to its position in Col-0. A model of intraspecific CNS variation is shown in figure 1*A*.

We investigated CNS present in the *A. thaliana* reference accession Col-0 identified by Haudry et al. (2013). These elements were identified through whole genome alignments of nine phylogenetically informative taxa within Brassicaceae resulting in a set of >60,000 CNS used in this study whose length distribution is shown in figure 1*C*. Regions were classified as CNS if they exhibited strong conservation across most investigated taxa. Conservation was measured using PhastCons (Siepel et al. 2005) resulting in CNS identified according to a likelihood score rather than presence or absence in a certain number of taxa. CNS were likely present in the majority of taxa as they required a high Phastcons score (>0.82) over at least seven nucleotides and did not include a region of more than twelve nucleotides with low PhastCons score (<0.55). As the Col-0 accession was the reference genotype for CNS identification, we did not investigate CNS present in other accessions which are absent in Col-0.

To investigate species level CNS variation, we first queried seven recently available long-read sequencing *de novo* genome assemblies for various *A. thaliana* accessions (tables S5 andS6, , Supplementary Material online; Jiao and Schneeberger 2020). As the set of query CNS were characterized for their presence in multiple different species across Brassicaceae spanning ∼32 million years of evolution (Edger et al. 2018), we expected little variation in these CNS at the species level. Each of these CNS were present in the *A. thaliana* reference accession Col-0. Indeed the vast majority of CNS are conserved in these seven other accessions. Of the 62,916 CNS investigated, we find an average of 209 (0.33%) and 951 (1.5%) CNS exhibit PAV and PosV, respectively, in each accession. However, querying seven accessions may be insufficient to capture the majority of the natural genetic variation occurring in these sequences as *A. thaliana* has a global distribution and at least nine definable genetic admixture groups (1001 Genomes Consortium 2016).

Therefore, we assembled the genomes of thirty *A. thaliana* accessions using a hybrid reference and de novo method (table S2, Supplementary Material online). This included assemblies for two accessions for which a long-read assembly was available for direct comparison (Ler-0 and Cvi-0). Our primary goal for comparing these long- and short-read assemblies was to assess the quality of the short-read genome assembly method and to identify high confidence PosV and PAV CNS. We find appreciable overlap in the PAV and PosV CNS identified between our assemblies and the long-read assemblies (tables S5 andS6, Supplementary Material online; fig. 1*B*). For example, roughly 84.9% of identified PosV CNS were shared between the long and short read assemblies for these two accessions. We observed lower (∼53.6%), but still significant (hypergeometric test *P*-value <2e–16) overlap in PAV CNS between the long- and short-read assemblies for these two accessions. This suggests that the overall quality of these short-read assemblies is sufficient for further analyses of these sequences.

Of the 62,916 CNS analyzed in the thirty short read genome assemblies, we find an average of 163 (0.26%; standard deviation = 41) and 910 (1.4%; standard deviation = 120) CNS exhibit PAV and PosV, respectively per accession (fig. 1*D*). These estimates are in line with those obtained using the long-read genome assemblies. Given the large number of CNS in the query set (62,916), this represents a definable class of sequence (>1,000 sequences per accession) with observable variation patterns. The subsequent analyses were performed on the larger set of 30 short-read assemblies.

Throughout the manuscript, CNS exhibiting PAV in at least one accession will be referred to as PAV CNS. A similar syntax will follow for CNS showing PosV in at least one accession. CNS in either of the aforementioned classes will be referred to as variable CNS, whilst those showing no variation are referred to as collinear CNS.

### Is CNS Variation Shared Among Accessions?

If PAV and PosV CNS occurred independently in each accession, we expect 4,567 and 21,699 different CNS to be lost and positionally variable, respectively in at least a single accession (, Supplementary Material online). In contrast to random expectation, we only observe 1,524 and 4,801 distinct CNS lost and positionally variable, respectively (figs. S1 andS2). About 56.6% of PAV and PosV CNS events are shared by multiple accessions (fig. S3). However, there is little overlap of PAV and PosV CNS. There are 118 CNS absent in at least one accession and positionally variable in at least one other accession (<10% of either set). This is not significantly different than expected by chance (hypergeometric test *P* = 0.4227782). We also investigated the correlation between PAV CNS, PosV CNS, and PAV gene counts. A stronger correlation exists between the number of PosV CNS and PAV genes (fig. S4; *R* = 0.81, *P*< 0.001) than PAV CNS (*R* = 0.62, *P*< 0.001). A weaker, yet still significant correlation, exists between PAV genes and PAV CNS (*R* = 0.36, *P*< 0.01).

Random subsampling of CNS analyzed in the 30 accessions indicates the majority of the natural common CNS variation is likely captured and is sufficient to investigate the functional consequences of this variation (figs. S1 andS2). Furthermore, there is strong observed overlap in variable CNS across accessions (figs. S3,S5, andS6), indicating there may be subclasses of CNS which are more likely to exhibit variation than others. This phenomenon is similar to certain types of gene families that often display copy-number variation (Rizzon et al. 2006; Dopman and Hartl 2007; Freeling 2009).

Lastly, similar to investigating gene PAV, sole consideration of a single reference genotype disables investigation of features completely absent in that reference. The *A. thaliana* reference genome Col-0 was used to identify CNS shared across Brassicaceae (Haudry et al. 2013). Therefore, there likely exist CNSs present in other accessions yet missing in the Col-0 reference. Though the identification of these sequences is beyond the scope of this study, one may posit the reference genotype Col-0 will contain comparable PAV and PosV figures relative to other accessions.

### Can CNS Structure Explain Environmental Associations Better than Population Structure?

Principal component analysis (PCA) was performed to examine similarities in CNS variation across accessions. PCA was performed separately using PAV CNS and PosV CNS as input. The first two principal components (PCs) for PAV CNS explained 10.8% and 8.29% of the total variance. Clustering accessions according to SNPs produced a topology similar to that constructed from PosV CNS information (fig. S7). Jointly using PAV and PosV CNS as clustering information produced topology similar to using PosV CNS alone.

We wanted to investigate whether clustering by CNS annotation aligns with bioclimatic variables obtained from WorldClim2 data (Fick and Hijmans 2017). Of the 19 bioclimatic variables obtained, strong correlations were observed (fig. S8). BIO1 (“annual average temperatures”) and “annual average precipitation” (BIO12) were selected as the best representatives of these variables as they were correlated with all other measures.

Before searching for associations, we must investigate the contribution of population structure to observed variation in CNS. Strong evidence exists for population structure and isolation by distance among accessions (1001 Genomes Consortium 2016; Nordborg et al. 2005; Platt et al. 2010; Hancock et al. 2011). Populations in close proximity exhibit greater similarity to neighbors than distant populations. Therefore, associations between the environment and genetic variants may simply reflect population structure. This relatedness must be accounted for in searches for such associations.

We evaluated the CNS PAV and PosV across samples relative to a neutral model of population structure generated from the first three axes of a whole genome SNP PCA (Zhang et al. 2010; RStudio Team 2020). This revealed that approximately 48% of the observed CNS PAV (Supp methods) could not be explained by the multiple linear model based predictor derived from PC1-3 of population structure alone. However, not all genes with CNS PAV were similar in this regard, and several hotspots of CNS variants with strong nonstructure driven association with climate were evident. These included several clusters on Chromosome 5 (19 genes) with an entirely consistent CNS PAV that were not evidently distributed in accordance with population structure but are associated with ambient temperature (fig. S9). Removing the CNS variants associated with these 19 genes from the analysis (as well as a single outlier sample: Gd-1) generated a CNS PAV PCA that could then be quite strongly predicted (PC1 *R*^2^=0.77) by the neutral multiple linear model. Introducing BIO1 (mean annual temperature) as an additional explanatory variable increased model *R*^2^ to 0.79, suggesting that beyond the core clusters of genes identified, an additional 22% of PAV was determined by processes beyond structure, likely due to experimental noise, error, or PCs 4+. PosV showed a closer relationship to overall population structure and while there was an evident relationship between the notable POSV: PC2 axis and BIO1, this was very similar to the relationship between population structure PC2 and Bioclim 1 (*R*^2^ = 0.20 and 0.23, respectively) confounding our ability to confidently assign the temperature association to PosV alone. In summary, we are unable to conclude CNS variants associate with environmental variables due to strong correlations with underlying population structure. However, this pattern does suggest that the majority of CNS variants are shared among *A. thaliana* populations.

### How Does CNS Variation Compare to Gene Content Variation?

Previous pan-genome studies devoted major efforts to characterize species level diversity in gene content and structural variants (Golicz et al. 2016; Gordon et al. 2017; Montenegro et al. 2017; Hübner et al. 2019). Most of these studies often do not fully assemble genomes for each individual of the species. Rather, they only assemble the sequence not present in the reference. It is challenging to identify positional conservation and rearrangements in non-reference individuals using these approaches. However, some previous pangenome studies (e.g.,*Brachypodium*; Gordon et al. 2017) have assembled full genomes but focused on only gene content variation. Our approach uses a hybrid reference guided and de novo assembly approach to obtain chromosome-scale sequences for each individual accession. This permits the analysis of PAV and PosV of both CNS and gene content.

Our analyses revealed CNS variation occurs at a much lower rate than genic PAV (fig. S10). This might imply purifying selection acts more strongly on noncoding regulatory regions than protein-coding genes. However, the noncoding regions investigated in this study are also present throughout Brassicaceae, biasing our annotations to CNS likely experiencing greater levels of purifying selection. We therefore analyzed the rate of gene PAV for genes present in all taxa used to identify CNS (Haudry et al. 2013). We find CNS variation occurs at a lower rate than gene PAV (9.87% and 20.13% variable in at least a single accession, respectively). The true rate of variation in functional noncoding regions may only be identified through complete annotation of functional *cis*-regulatory regions, a difficult feat relative to the annotation of coding regions. Thus, it is imperative that future efforts identify lineage-specific CNS to assess the full scope of regulatory variation that exists at the species level.

### What Is the Length Distribution of Variable CNS?

The distribution of the lengths of CNS was investigated (fig. S11). CNS retaining their syntenic position in every accession (collinear CNS) have a length distribution similar to that of all CNS in the reference accession. PAV CNS on average have a longer length (in base-pairs) than collinear CNS (collinear average = 39.84, PAV average = 44.41, *KS* test *P*< 2.2e^−16^). The PAV CNS length distribution appears slightly bimodal (fig. S11). PosV CNS are much shorter on average than either collinear or PAV CNS (PosV average = 18.97).

### *What Is the Distribution of CNS Movement Events*?

**Fig. 2.**
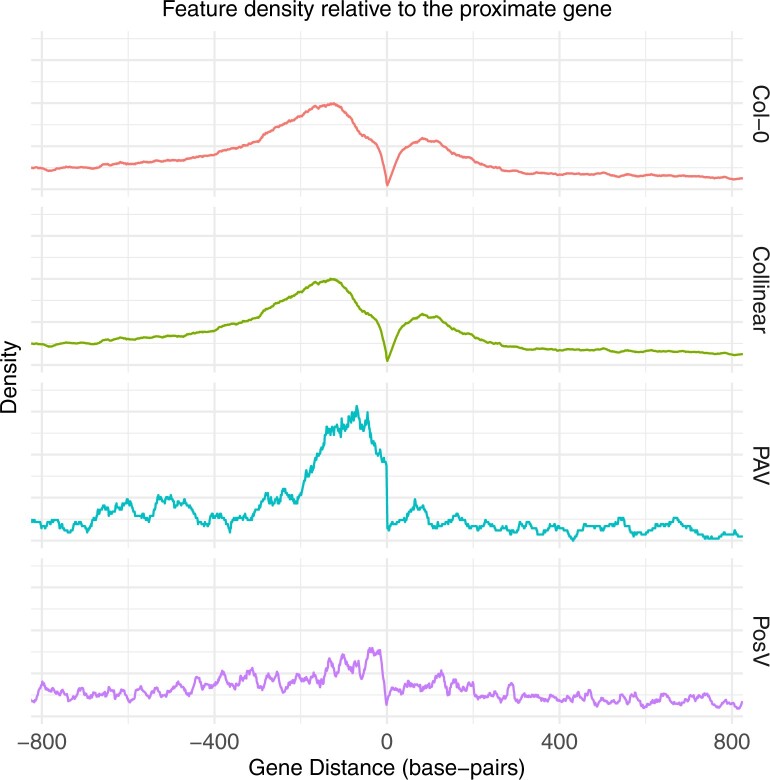
Distributions of different CNS features relative to their proximate gene are shown, where the x-coordinate zero represents the location of the proximate gene. The top panel (red) shows all CNS in the reference accession Col-0. The second panel (green) shows across all accessions the distribution of CNS that remain in the same syntenic position as the reference accession (collinear CNS). The next panel (blue) shows the position in the reference accession where PAV CNS are located, i.e. the position in Col-0 where CNS display PAV in any of the thirty accessions. The fourth row (purple) shows the position of PosV CNS across all accessions, i.e. the location to which these CNS “moved”.

CNS distance to their proximate gene was investigated. Figure 2 shows the distance of CNS to their proximate genes across the different classes of CNS. The largest concentration of CNS is intergenic and close to genes in the genome (37.17% of CNS ± 500 bp of and between transcriptional start or termination sites). There is a reduction in the concentration of CNS around the nearest gene for PosV CNS, relative to CNS in accessions that retain their syntenic position in Col-0. Position relative to the proximate gene does not seem to predispose a CNS from exhibiting PAV.

We tested the hypothesis that PosV CNS occur closer to genes than randomly expected by permuting the location of PosV CNS randomly across the genome (fig. S12). We found PosV CNS occur closer to genes than if they randomly move around the genome (KS-test *P*< 0.01). However we also compared their distribution to positionally conserved (collinear) CNS. This revealed PosV CNS occur further from genes than collinear CNS. Though PosV CNS occurrence closer to genes than random may imply functional constraints, their increased distance relative to collinear CNS might be a result of reduced selective constraints and/or simply an outcome of the transposition mechanism. For example, the movement of a CNS by TE must occur at specific intergenic sites to minimize negative impacts to nearby gene functions (Zhang et al. 2020).

### Are Variable CNS Associated with Accessible Chromatin?

We performed Assay for Transposase-Accessible Chromatin sequencing (ATAC-seq) in leaf tissue for eighteen accessions, including the reference accession Col-0. This method identifies genomic regions accessible by a Tn5 transposase (Buenrostro et al. 2015; Bajic et al. 2017; Lu et al. 2017), and such regions of accessible chromatin are often associated with *cis*-regulatory DNA elements and transcription factor binding (Galli et al. 2018; Lu et al. 2019; Ricci et al. 2019; Parvathaneni et al. 2020). We utilized a protocol which combines fluorescence-activated nuclei sorting and ATAC-seq (FANS-ATAC-seq; Lu et al. 2017). As we hypothesize CNS are regulatory sequences, we expect that CNS will be enriched within regions of accessible chromatin. ATAC-seq reads were aligned to their respective genome, and peaks, regions of statistically enriched clusters of sequencing reads that are indicative of accessible chromatin, were identified. Collinear CNS demonstrated much stronger overlap with ATAC peaks than expected by chance (average fold-enrichment = 3.086; table S1, Supplementary Material online). Three accessions were removed from this analysis due to poor library quality (table S1, Supplementary Material online). Across all accessions, an average of 14.03% of CNS annotations overlapped chromatin accessible regions. Furthermore, we tested whether CNS which deviate from their position in the Col-0 accession (PosV CNS) still overlapped ATAC peaks. The average fold enrichment for PosV CNS across all accessions was 1.37. The average percent overlap was 6.72%, lower than observed for collinear CNS. Permutation analysis demonstrated this overlap was unlikely a result of the shorter length of PosV CNS (table S1).

**Fig. 3.**
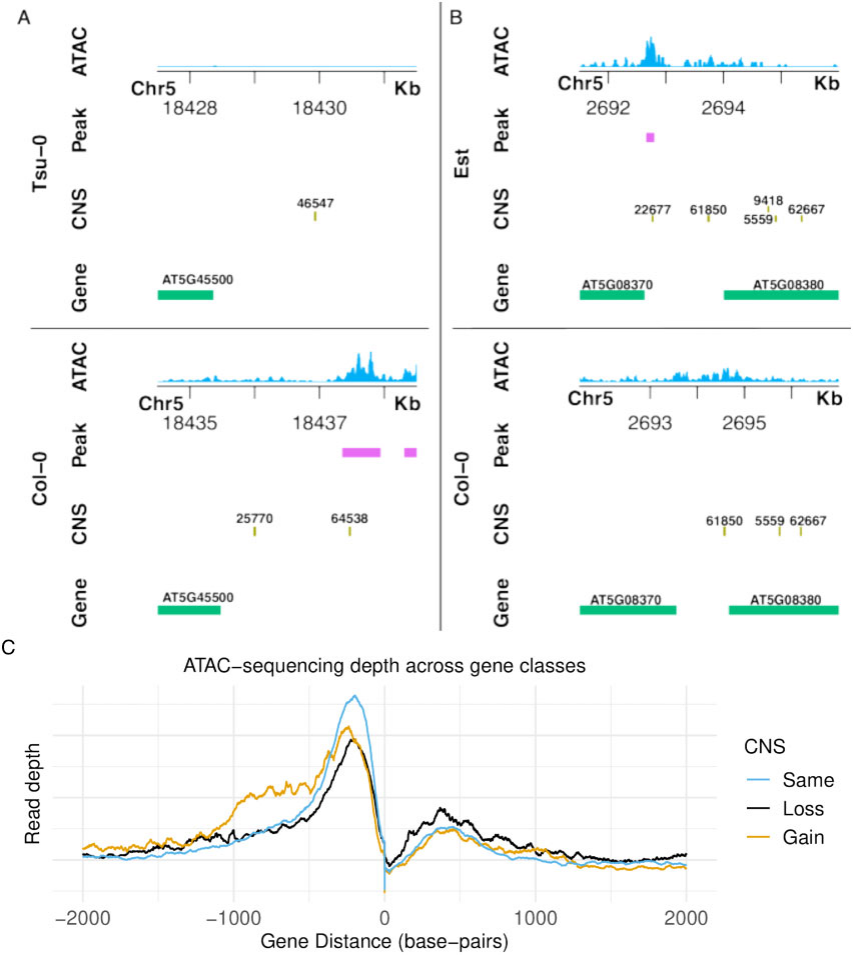
Genome browser tracks are shown for two different syntenic regions. (*A*) Shows loss of CNS 64538 and 25770 associated with loss of ATAC-seq peaks downstream the locus AT5G45500. Part (*B*) demonstrates a gain of CNS 22677 associated with the gain of a CNS peak upstream the locus AT5G08370. Coordinates are relative to each genome assembly. Thus orthologous sequences may have different coordinates due to insertions or deletions occurring in upstream coordinates. (*C*) ATAC-sequencing depth is shown surrounding orthologous genes with either the same (blue), less (black), or more (orange) CNS associated with them relative to the reference accession Col-0.”

Strong evidence of CNS overlapping signatures of accessible chromatin has been reported previously (Zhang et al. 2012; Van de Velde et al. 2014; Lai et al. 2017; Zhao et al. 2018; Lu et al. 2019). In each case, the set of CNS queried was different, with estimates ranging from 14% to 48% of CNS overlapping signatures of accessible chromatin (table S3). The percentage reported here, 14%, is in line with previous estimates. In figure 3, we demonstrate an instance where CNS loss is associated with loss of accessible chromatin in a given accession. Additionally, we show a novel CNS insertion in an accession associated with an accession-specific accessible chromatin region. Figure 3*C* provides some additional support for an association between accessibility and CNS presence or absence, with an increase in accessibility upstream of genes associated with a greater number of CNS compared to the reference accession.

Though there is significant overlap between PosV CNS and accessible chromatin, the majority of PosV CNS do not overlap ATAC peaks. The true proportion of putatively functional PosV CNS is likely greater than observed here, potentially because the ATAC sequencing was performed at a single time point in a single organ under normal growing conditions. As CNS are hypothesized to perform regulatory functions, their binding partner may require distinct spatial–temporal context and/or environmental stimuli to activate. Given bulked tissue samples and homogenous environmental stimuli were sampled for the ATAC-seq data, it is unlikely every PosV CNS which may exist in regions of accessible chromatin under different conditions will be identified. In addition to responding to distinct environmental stimuli, regulatory functions of some CNS may also be cell/tissue/organ or developmental stage specific, further lending to their absence in regions of accessible chromatin observed here. Lastly, PosV CNS and ATAC peak overlap was lower than that of collinear CNS. PosV CNS may act as adaptive sequences, changing over shorter evolutionary distances, similar to certain classes of genes that exhibit higher transposition and duplication rates (Rizzon et al. 2006; Freeling et al. 2008; Edger and Pires 2009). They may be involved in specific stress responses and therefore may not demonstrate overlap with accessible chromatin in healthy leaves. However, future work is required to assess specifically whether PosV CNS exhibit these behaviors.

Alexandre and coworkers previously investigated variation in signatures of accessible chromatin and sequence diversity of differentially accessible regions across five diverse *A. thaliana* accessions (Alexandre et al. 2018). They discovered ∼15% of accessible chromatin regions differed across the five accessions, with a minority of those sites displaying sequence divergence. However, mapping data from non-reference genotypes to a reference genome may result in reference mapping bias (Degner et al. 2009). By assembling separate genomes for each accession, we mitigate this bias. We add to the findings of Alexandre and coworkers by characterizing sequence diversity directly on a larger panel of thirty accessions, focused on a CNS set consisting of more than 3 Mb of sequence, and discover significant relationships between variable sequence and regions of accessible chromatin.

It should be noted that previous studies posited accessible chromatin region differences between *A. thaliana* cell types were primarily quantitative rather than qualitative (Maher et al. 2018). Perhaps PosV CNS not overlapping with accessible chromatin align with this trend and exhibit low signatures, rather than absence, of accessible chromatin below our detection threshold. This is worth investigating in the future, especially as single cell accessible chromatin data becomes available.

### Is CNS Loss-and-Gain Associated with Gene Expression Differences?

RNA-sequencing (RNA-seq) data were analyzed for four of the accessions investigated in this study to identify differentially expressed genes in leaf tissue. Each comparison was between an accession and Col-0 (Gan et al. 2011). Genes with a greater number of CNS associated with them in a given accession were more likely to be upregulated in that accession (table 1). Genes with a lower number of CNS associated with them in a given accession were more likely to be downregulated in that accession (table 1). Genes without CNS changes relative to Col-0 were significantly underrepresented for differentially expressed genes (table 1). This demonstrates a significant association between changes in *cis*-regulatory sequence and divergent expression, a phenomenon also demonstrated across populations of stickleback fish (Verta and Jones 2019). If the true ratio of activator binding sites to repressor binding sites were equal, we would expect no enrichment for differentially expressed genes for those gaining or losing CNS. Our results suggest CNS variation tilts towards a greater number or activity of *cis*-acting activator (enhancing expression) binding sites.

**Table 1.** Tests for over and under representation of differentially expressed genes in CNS with greater than (CNS Gain), less than (CNS Loss), or no change (No CNS Change) relative to Col-0. Numbers in parenthesis refer to the percent of the gene types (CNS Gain, CNS Loss, No CNS Change) which are up or down regulated.

Gene type	Average number of upregulated genes per accession (%)	*P*value (hypergeometric test)	Average number of downregulated genes per accession(%)	*P*value (hypergeometric test)
CNS gain	270.25 (6.30)	<1 × 10^–10^	112 (2.61)	<1 × 10^–10^
CNS loss	49.5 (1.86)	<1 × 10^–10^	203.25 (11.357)	<1 × 10^–10^
No CNS change	404.75 (2.212)	<1 × 10^–10^	631.5 (3.380)	<1 × 10^–10^

Underrepresented.

Overrepresented.

### Are Variable CNS Enriched with Certain Binding Motifs?

PAV and PosV CNS were searched for enriched motifs with the program HOMER (Heinz et al. 2010). The set of all PAV and PosV CNS were tested separately. Motifs for the binding targets of several stress responsive transcription factor families were enriched. Specifically, in the set of PosV CNS sequences, the binding motifs of *MYB113*, *C2H2*, *ABF3*, *HSF21*, *WRKY8*, and *CBF4* were enriched. For PAV CNS, *WRKY50*, *RAV1*, and *Dof2* motifs were enriched. The global pattern for enriched motifs were for stress responsive elements. Given there are environmental differences experienced by these different accessions, we hypothesize differences in regulatory patterns may govern an accession’s stress response. Most of these enriched motifs are found in several PosV CNS leading us to hypothesize widespread rewiring of stress responsive pathways has occurred across *Arabidopsis* accessions.

As CNS are enriched with stress responsive motifs, we were curious if their associated genes demonstrated similar enrichments. Genes near PosV CNS which overlapped ATAC peaks were associated with various GO terms associated with phytohormones and response to various abiotic stresses, including “response to water deprivation,” were highly enriched (FDR P-value = 7.51e-09). Interestingly, among the genes associated with PosV CNS are a set of 31 genes that have been previously associated with abiotic stress tolerance by modifying abscisic acid (ABA) phytohormone levels (FDR *P* = 8.66e–08) (Sah et al. 2016). In addition, there are nineteen genes in the ethylene signaling pathway (FDR *P*< 0.01). This pathway is also associated with stress response in plants (Müller and Munné-Bosch 2015). Genes near PAV CNS were enriched for a large number of GO terms associated with “regulation,” most notably associated with “developmental process” (FDR *P* = 2.41e–10) and “anatomical structure development” (FDR *P* = 1.08e–10), including “shoot system” (FDR *P* = 5.23e–6) and “root development” (FDR *P* = 3.48e–5), but also “response to abiotic stimulus” (FDR *P* = 0.00044). PAV CNS are more highly enriched for development specific than stress related GO terms. However, PosV CNS were also enriched with “developmental process” (FDR *P* = 1.2e–17) and “anatomical structure development” (FDR *P* = 7.44e-15), including “shoot system (FDR *P* = 3.18e-9) and ‘root’ development” (FDR *P* = 0.0057). The overlap in stress response and developmental GO terms may likely be that the two aforementioned phytohormones (ABA and ethylene) are long known to be involved in both biological processes.

### Are CNS Changes Associated with Altered Selective Constraints?

As mentioned earlier, each CNS is associated with a gene in the Col-0 reference genome. Therefore, we can track the orthologous genes in each accession and determine if the genes in accessions which lose CNS exhibit signatures of positive or negative selection compared to those which have retained the CNS, including the Col-0 reference. We assigned PosV CNS to their proximate gene. Strandedness or relative position (upstream or downstream) were not considered for assigning PosV CNS to a gene, purely distance to the nearest gene. We searched for signatures of selection in regions experiencing CNS loss, gain, or neither.

PiN/PiS ratio was calculated. For every gene with an ortholog in all thirty accessions (*n* = 20,096), we grouped those which lost a CNS, those which gained a CNS, and those with an equivalent number of CNS associated with them relative the Col-0 reference. For an equivalent comparison between these three classes, we selected only orthologs with at least two accessions in every class (*n* = 564). The groups were aligned separately, however the Col-0 ortholog was included as the outgroup for every alignment. We observe no obvious differences between the various gene classes (fig. S13).

We also explored nucleotide diversity in these protein coding regions. The lowest mean diversity was observed for genes associated with an equivalent number of CNS relative the Col-0 reference (mean = 0.0133). Though genes with fewer and more CNS associated with them relative to the reference exhibit higher diversity (mean = 0.0179 and 0.0163, respectively), these differences are not large. There also may be several contributing factors to observed differences. Elevated rates of nucleotide diversity in protein coding regions may reflect relaxed selective constraints in polymorphic CNS regions. However, it may also simply reflect stochastic differences in mutation rates, as nucleotide diversity values vary across the genome. It is important to consider alternate hypotheses in the future. Overall, we do not find global signatures of selection in regions experiencing CNS variation.

### Are There Relationships between CNS Class and Repeats or Gene Duplicates?

**Fig. 4.**
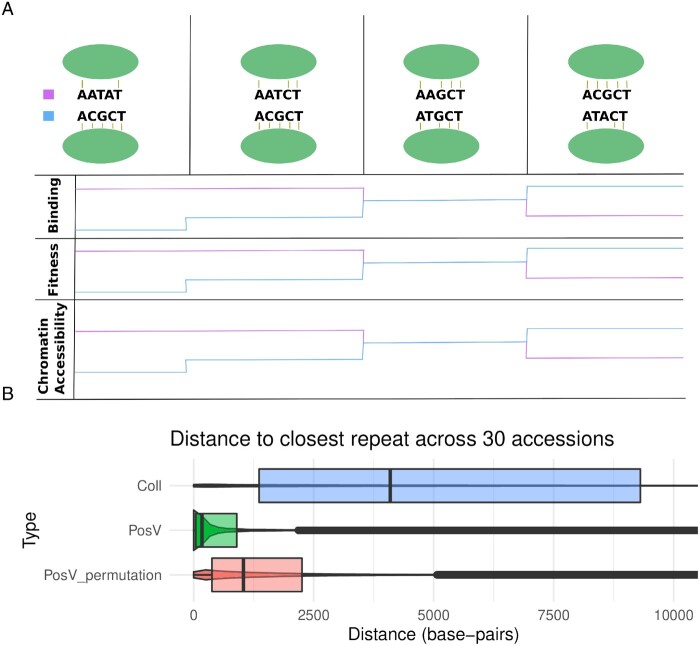
Hypothesized changes to transcription factor binding, fitness, and chromatin accessibility around two regions of DNA: the new PosV CNS location (top, blue), and the ancestral position as observed in Col-0 (bottom, pink). Each column in the first row depicts a snapshot of the state of the two CNS locations as time progresses along the x-axis. The hypothesized changes to TF binding, organism fitness, and chromatin accessibility align with the different time points, the breaks of which indicate a mutation occurring in both sequences.

We investigated the proximity of repeats for different classes of CNS. We extracted repeat annotations generated by MAKER2 (Holt and Yandell 2011). Figure 4*B* demonstrates a clear bias in colocalization between PosV CNS and maker-annotated repeats relative to collinear CNS (*KS* test *P*< 2.2 × 10^−16^). The trend for each accession separately is consistent (fig. S14). This colocalization between PosV CNS and TEs may underlie a possible mechanism of CNS movement. We theorize, as shown previously for regulatory elements (Bourque et al. 2008; Schmidt et al. 2012; Zhao et al. 2018; Lu et al. 2019), transposing TEs may incorporate CNS during transposition and, thus, may distribute a particular CNS around the genome. However, it is important to consider how repetitive regions may impact the identification of CNS. This should be investigated in greater detail in the future.

Lastly, we compared CNS content for different classes of gene duplications (fig. S15). Considering only genes with a CNS associated with them, tandem duplicates had the fewest CNS associated with them (mean CNS count = 0.2442). This may be an artifact of CNS identification algorithms which struggle with tandem repeats. Alternatively it may be that genes which tend to be tandemly duplicated, such as TIR-NB-LRR disease resistance genes (Freeling 2008), have fewer CNS. Genes without any duplicate in the genome (mean CNS count = 0.7064) had less CNS associated with them than genes with a duplicate pair dating back to the most recent whole genome duplication (At-alpha) shared by *A. thaliana* (mean CNS count = 0.9824) (Edger et al. 2018). This observation is consistent with previous studies; genes associated with CNS were more likely to be retained as duplicate pairs through diploidization potentially due to gene dosage constraints (Birchler and Veitia 2010) or simply that these genes have long subfunctionalizable regulatory regions (Freeling et al. 2015), or both explanations might be correct.

## Discussion

This study is, to our knowledge, the first genome-wide survey of CNS PAV and PosV at the species level in plants. The rate of variable CNS, while small compared to variable genes, is higher in *A. thaliana* than we expected considering these sequences exhibit broad conservation across Brassicaceae. However, the numbers reported here are likely underestimates of variable functional noncoding sequences given that our CNS set is heavily skewed towards those likely under stronger purifying selection. These CNS were identified by aligning multiple Brassicaceae genomes spanning millions of years of evolution (Haudry et al. 2013). Thus, new methods are needed to identify the full complement of functional regulatory sequences that are lineage and even species specific. Furthermore, future studies with more diverse ATAC-seq libraries (e.g., tissue/cell specific, various abiotic stresses) are needed to functionally characterize both the variable and conserved CNS in *Arabidopsis*.

How is it that nearly 1,000 PosV CNS are at different loci in distinct accessions? We present two non-mutually exclusive hypotheses (fig. 4). First, we propose a *de novo* origin hypothesis. We find the distribution of PosV CNS lengths to be noticeably shorter than the length distribution of all CNS (fig. S11). PosV CNS are often less than twenty base-pairs in length. Therefore, perhaps the majority of the CNS sequence already exists in alternate loci, and only a few base-pair changes are needed to convert an existing background sequence to a CNS (fig. 4*A*). A DNA sequence, which is very similar to a binding motif, may experience partial binding of a given transcription factor. This may be the selective pressure required to convert, or rather select for, beneficial mutations on the existing sequence to further strengthen that TF’s binding.

Second, the movement of regulatory elements may involve transposable elements (TEs) as shown previously (Bourque et al. 2008; Schmidt et al. 2012; Zhao et al. 2018; Lu et al. 2019). Indeed, we observe strong bias with respect to the colocalization of PosV CNS and maker annotated repeats relative to collinear CNS (fig. 4*B*;  fig. S14; Coll CNS median = 4096 bp, PosV CNS median = 173 bp). Shorter CNS would be more likely to remain intact during transposition. These hypotheses, de novo origin and TE transposition, are not mutually exclusive, and both may explain how PosV CNS arise at non-reference locations and skewed towards shorter lengths relative to collinear CNS (fig. S11).

Evolution of enhancer elements has been well studied in mammals (Villar et al. 2015; Emera et al. 2016). These studies revealed thousands of lineage-specific enhancer elements have evolved across mammals and often occur in “ancient” DNA that is significantly under enriched for flanking repetitive elements. This suggests that lineage-specific enhancer elements may arise through de novo origins via random mutations, in line with one of our hypotheses. Additionally, a few studies in *Drosophila* demonstrated de novo origins of TFBS (6–8bp) can occur on the order of 10^3^–10^6^ years under a model of neutral evolution (Stone and Wray 2001; Berg et al. 2004) which is within the divergence time (10^4^–10^5^ years) among *A. thaliana* accessions (1001 Genomes Consortium 2016). Additional support for our hypothesis can be found in a model of binding site evolution proposed by Mustonen and Lässig (2005). According to their model, selective strength on random mutations depends upon the mutation’s effect on the binding strength of its associated transcription factor. Therefore, selection for partial transcription factor binding may drive sequence conversion from partial to full CNS sequence as shown in figure 4*A*.

Lastly, we provide evidence that positionally variable CNS retain significant associations with regions of accessible chromatin. Additional evidence for the function of PosV CNS, such as the effect of CNS change on the expression of specific genes, should be the focus of future studies in *A. thaliana*. This may need to involve genome editing of target CNS to assess its direct impact on gene expression and phenotype. We hypothesize PAV and shuffling of existing CNS at the population level serves as a mechanism to navigate the evolutionary landscape. Much like other structural variants, many CNS variants may be selected against. However, beneficial CNS variation may undergo positive selection to fuel fitness improvements. Future studies should also investigate what proportion of shared CNS variants between populations are due to convergent or parallel evolution driven by selection to adapt to similar environments.

## Data Availability

ATAC sequencing data are available on the GEO under accession code GSE164159. Genome assemblies, gene annotations, and CNS annotations for each accession are deposited on Dryad (https://doi.org/10.5061/dryad.pzgmsbcfv; last accessed February 16, 2021). The CNS annotations for the reference accession were taken from Haudry et al. (2013).

## Author Contributions

All authors performed the research and/or analyzed data; A.E.Y and P.P.E. drafted the manuscript. All authors suggested experiments, reviewed and edited the manuscript.

## Supplementary Material

msab042_Supplementary_Data
