# Supplementary material for: Evolution of Conserved Noncoding Sequences in Arabidopsis thaliana
Source: Mol Biol Evol. 2021 Feb 10;38(7):2692–703. doi: 10.1093/molbev/msab042 (PMC8233505; doi:10.1093/molbev/msab042)
Supplement: msab042_Supplementary_Data [file msab042_supplementary_data.zip › Yocca_CNS_Supp_Fig_21_01_27.pdf]

Title: Evolution of conserved noncoding sequences in *Arabidopsis thaliana*

Alan E. Yocca[1,2], Zefu Lu[3], Robert J. Schmitz[3], Michael Freeling[4], Patrick P. Edger[2,5]

1. Department of Plant Biology, Michigan State University, 612 Wilson Rd. East Lansing MI 48823
2. Department of Horticulture, Michigan State University, 1066 Bogue St. East Lansing, MI 48824
3. Department of Genetics, University of Georgia, 120 Green Street, Athens, GA 30602-7223
4. Department of Plant and Microbial Biology, University of California, 111 Koshland Hall, Berkeley, CA 94720
5. Ecology, Evolutionary Biology and Behavior, Michigan State University, East Lansing, MI, USA 48824

Figure S1:

Figure S1 compares subsampling results for PAV CNS against random CNS sampling. Each point along the x-axis corresponds to the average unique number of PAV CNS across 1,000 random accession samples of size X (changing along the x-axis) with error bars representing +/- 1 standard deviation from the mean of the samples. The PAV_permutation line was created by taking a random sample of CNS instead of a sample of the annotated PAV CNS in each accession.

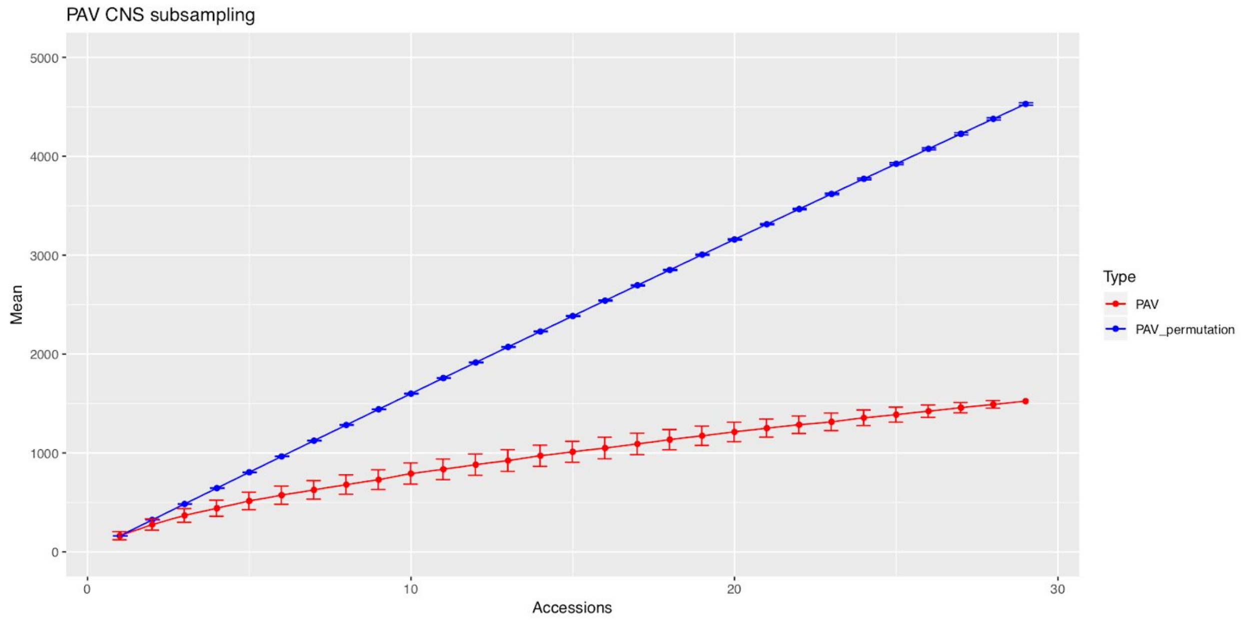

Figure S2:

Figure S2 compares subsampling results for PosV CNS against random CNS sampling. Each point along the x-axis corresponds to the average unique number of PosV CNS across 1,000 random accession samples of size X (changing along the x-axis) with error bars representing +/- 1 standard deviation from the mean of the samples. The PosV_permutation line was created by taking a random sample of CNS instead of a sample of the annotated PosV CNS in each accession.

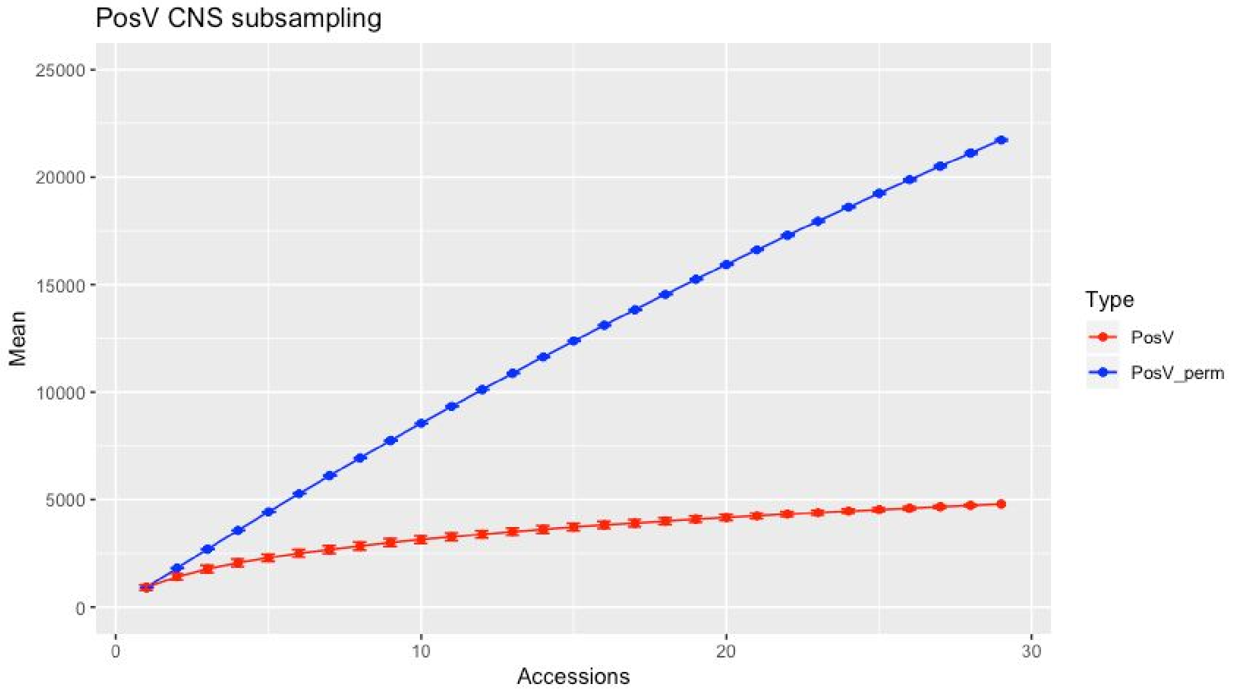

Figure S3

Figure S3 shows a histogram of the number of accessions variable CNS are present in. Of 6,207 total variants (PAV and PosV CNS), only 2,692 (43.4%) are specific to a single accession. The curve displays the cumulative distribution.

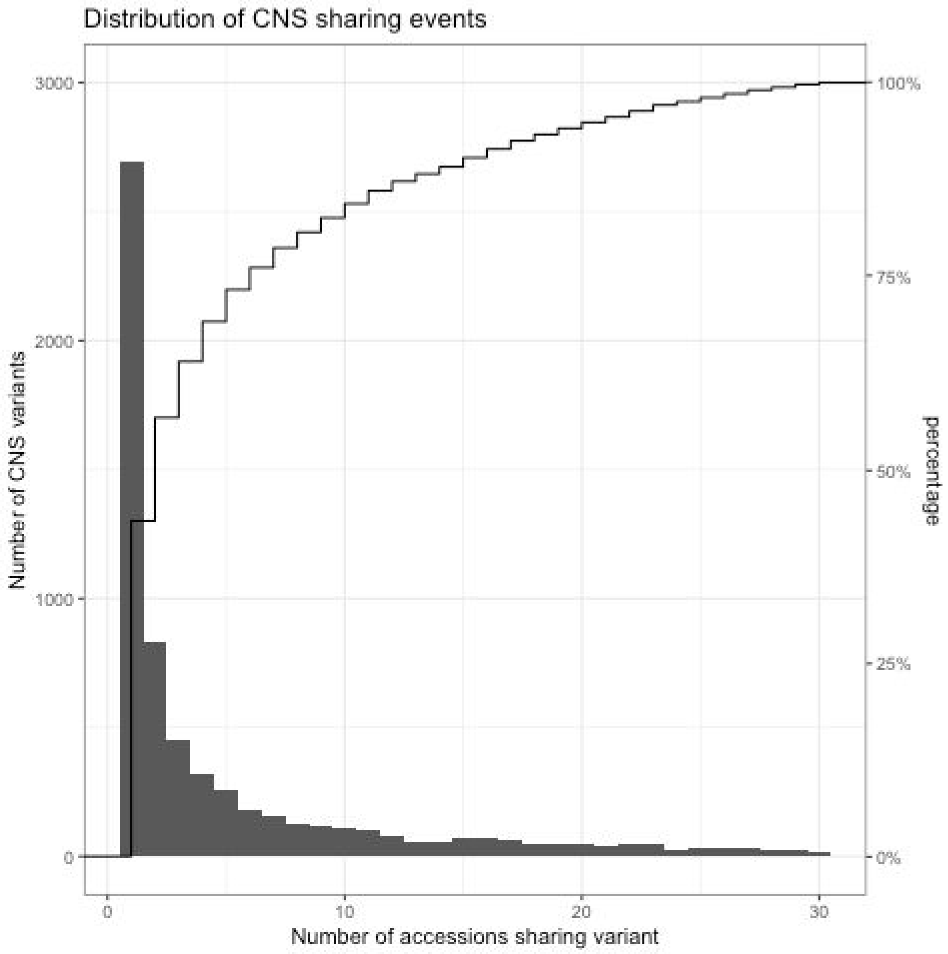

Figure S4:

Figure S4 correlates the number of PAV CNS with the number of PosV CNS and PAV genes for each accession, excluding Gd-1 (333 PAV CNS; 868 PosV CNS; 3329 PAV genes). This figure shows a significant Pearson's correlation value of 0.62 (p-value < 0.001). Removing Gd-1 resulted in much stronger correlations between these values.

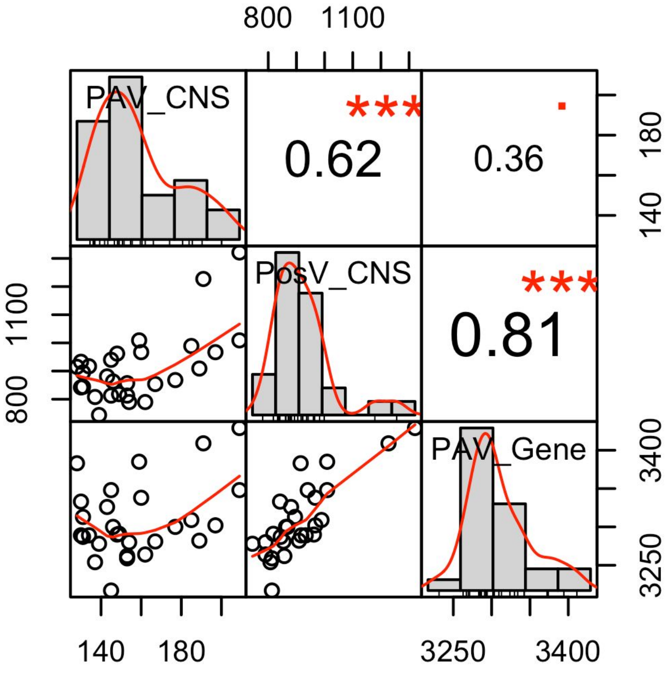

Figure S5:

Figure S5 is a plot generated by the R package UpSetR (Conway et al. 2017). It shows the highest overlapping pairwise comparisons for PAV CNS across accessions.

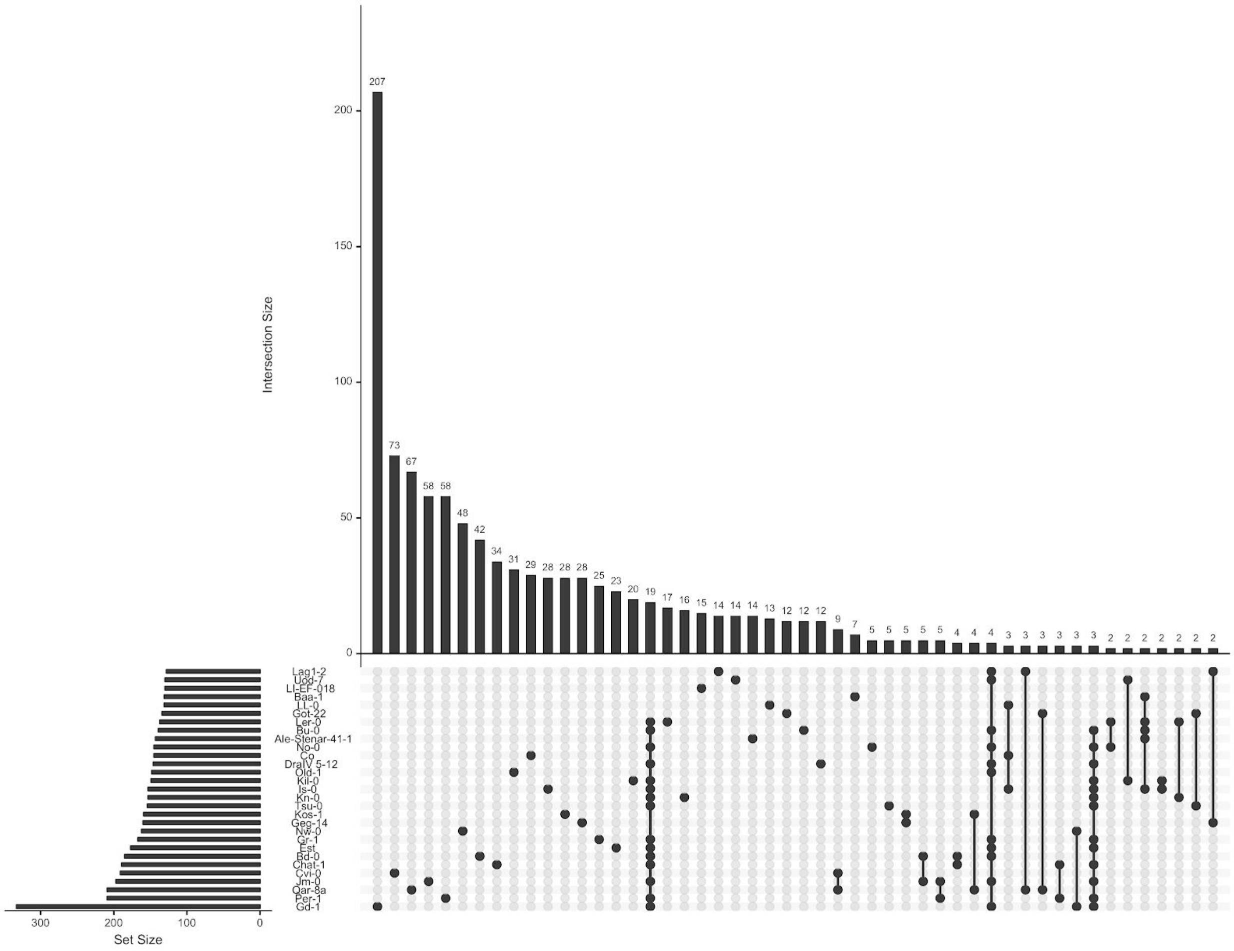

Figure S6:

Figure S6 is a plot generated by the R package UpSetR (Conway et al. 2017). It shows the highest overlapping pairwise comparisons for PosV CNS across accessions.

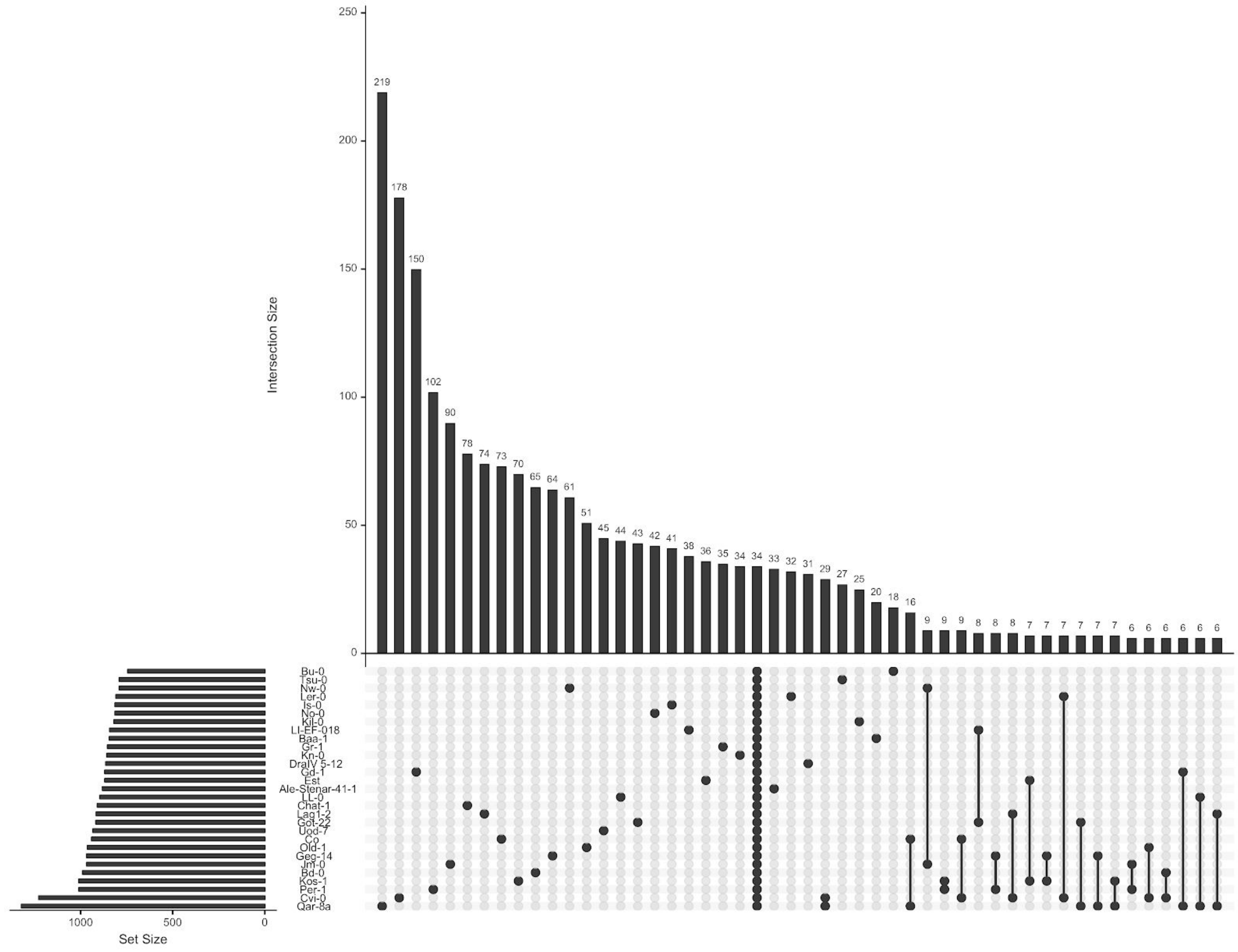

Figure S7:

Figure S7 shows the first two principal components for: (A) PAV CNS, (B) PosV CNS, (C) SNP information, and (D) PAV and PosV CNS considered jointly. The amount of variation explained by the first two principal components is labeled on the axis labels. Accessions are colored according to their admixture groups as defined by the 1001 Genomes Consortium (1001 Genomes Consortium 2016).

A

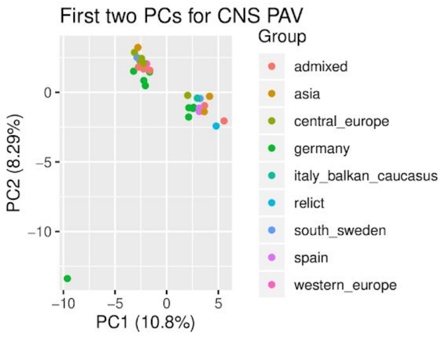

B

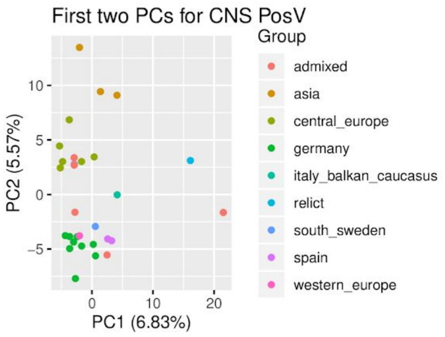

C

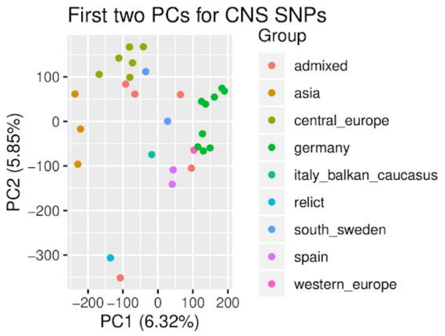

D

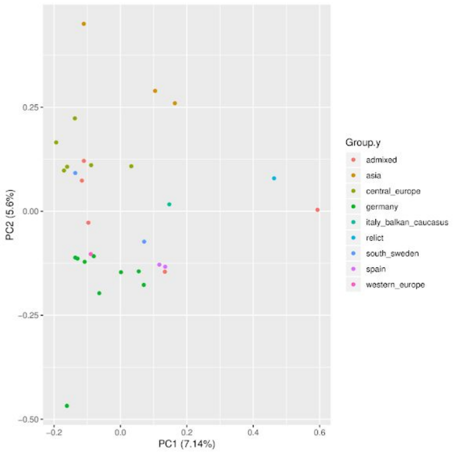

Figure S8:

Figure S8 shows the pairwise correlations of all 19 bioclimatic variables used in this study. We observe strong correlations for every measure involving temperature and precipitation. This is highlighted by the red rectangles drawn across the specific transects. Therefore, we decided to investigate only BIO1 (annual average temperatures) to capture most of the variation in these variables. This plot was generated using the corrplot R package (Taiyun Wei And 2017).

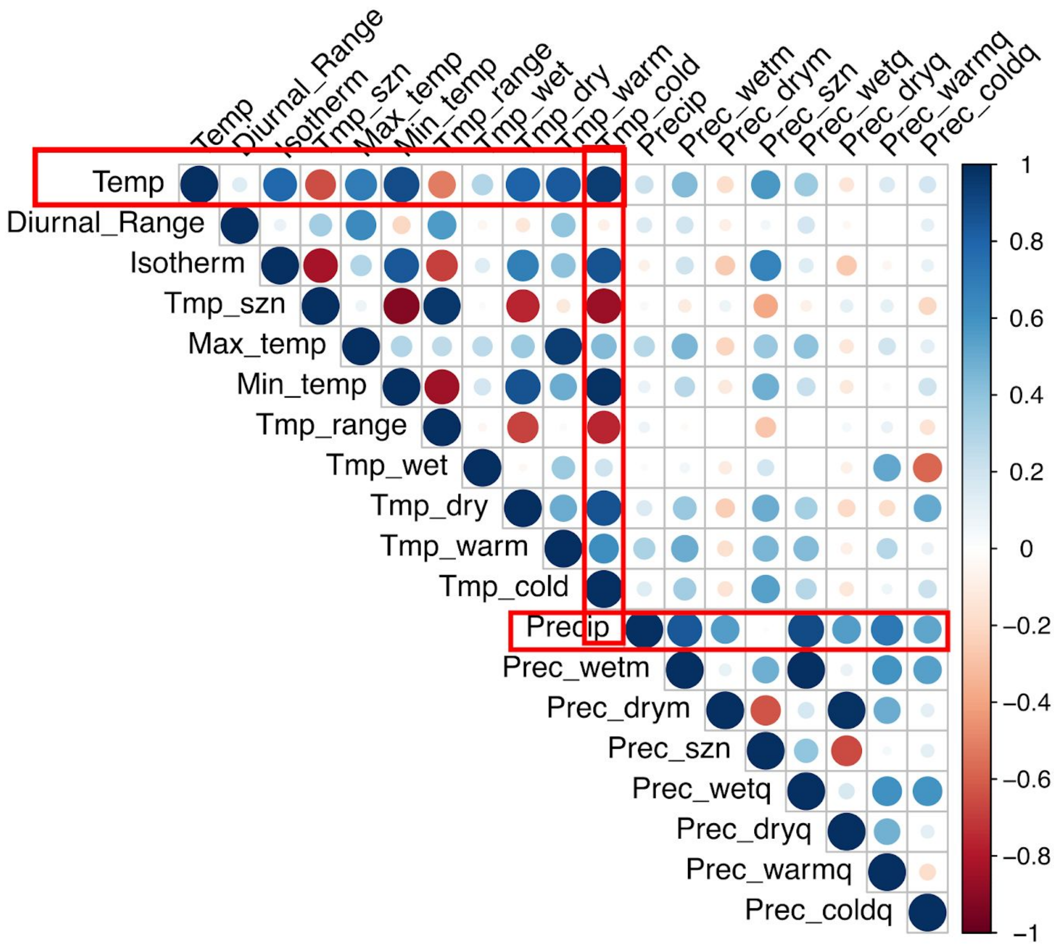

Figure S9

Hierarchical clustering of 30 *A. thaliana* lines (x-axis) by CNS presence absence variation (y-axis) is strongly driven by a subset of loci on chromosome 5 that show an entirely correlated trend in PAV (black arrow).

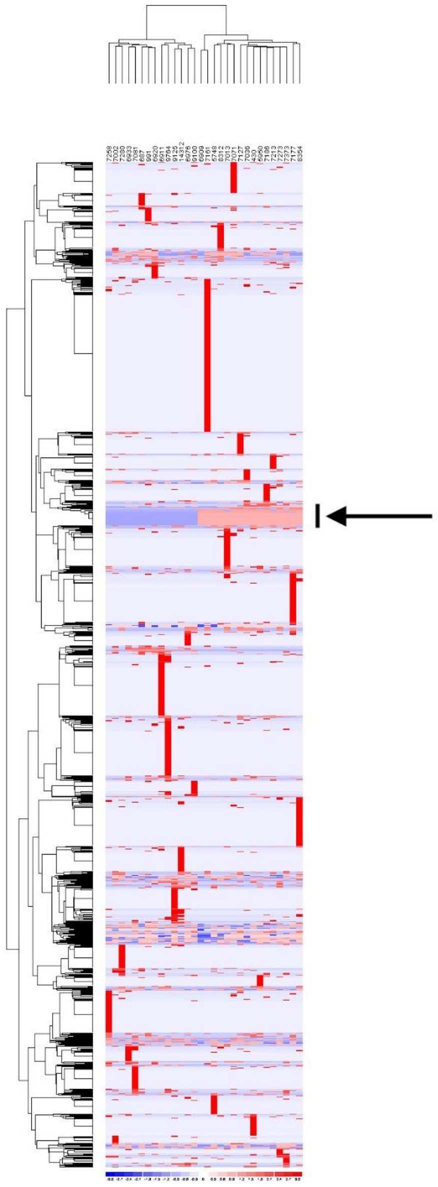

Figure S10:

Figure S10 compares subsampling results for PosV CNS, PAV CNS, PAV genes, and cruciferous PAV genes. For each point along the x-axis, 1,000 random samples of accessions of size X (increasing along the x-axis) were taken. For each sample, the proportion of unique polymorphism events was calculated. The points plotted are the averages across all 1,000 samples with error bars representing +/- 1 standard deviation from the mean of the samples. Cruciferous genes (Cru_Gene) were identified by orthofinder2 (Emms D. M. 2018) using the same set of query species used to identify CNS in Haudry et. al. 2013 (Supplementary Methods). Importantly, gene PAV was defined as genes present in the reference accession Col-0 and absent in at least a single other accession to keep consistent with our definition of PAV CNS.

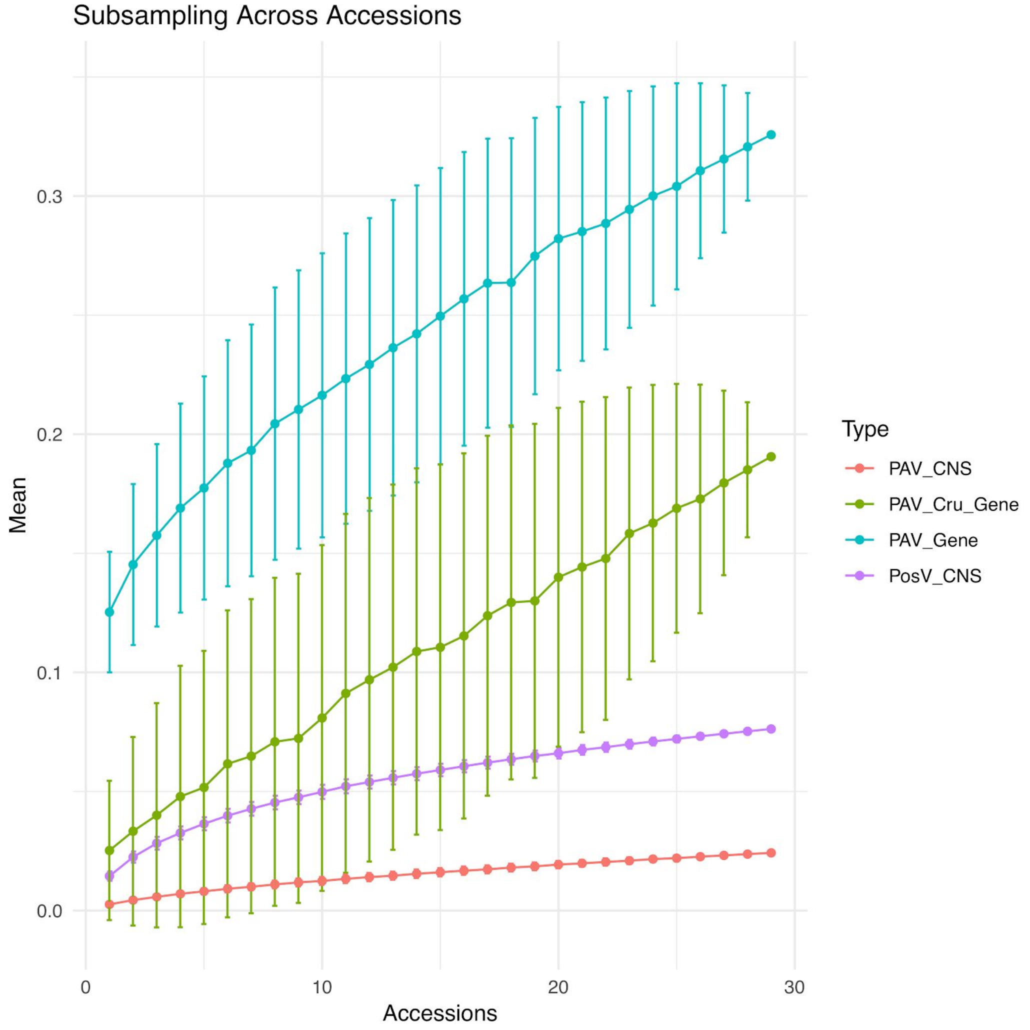

Figure S11:

Figure S11 compares the distribution of lengths of PosV CNS to the length distribution of all CNS. Strikingly, PosV CNS are shorter than expected by chance.

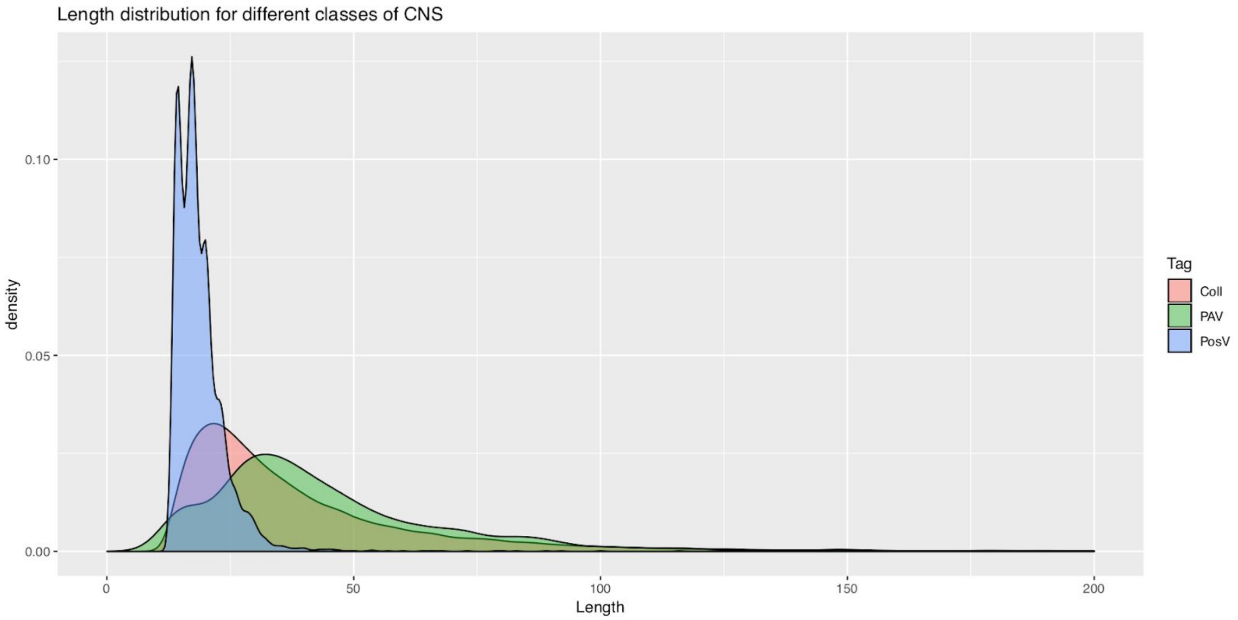

Figure S12:

Figure S12 compares the distribution of distances of PosV CNS (PosV) to the distribution of collinear CNS distances to proximate genes across the genome (Coll) and random locations (Permutation). PosV CNS demonstrate insertional bias closer to genes than random, yet further from genes compared to collinear CNS. Distance is measured in base-pairs. All distributions are significantly different from each other within each accession (p-value < 0.01, Kolmogorov-Smirnov test). Note for accession Col-0, PosV CNS are those found in our independent assembly of this accession and likely reflect false positives.

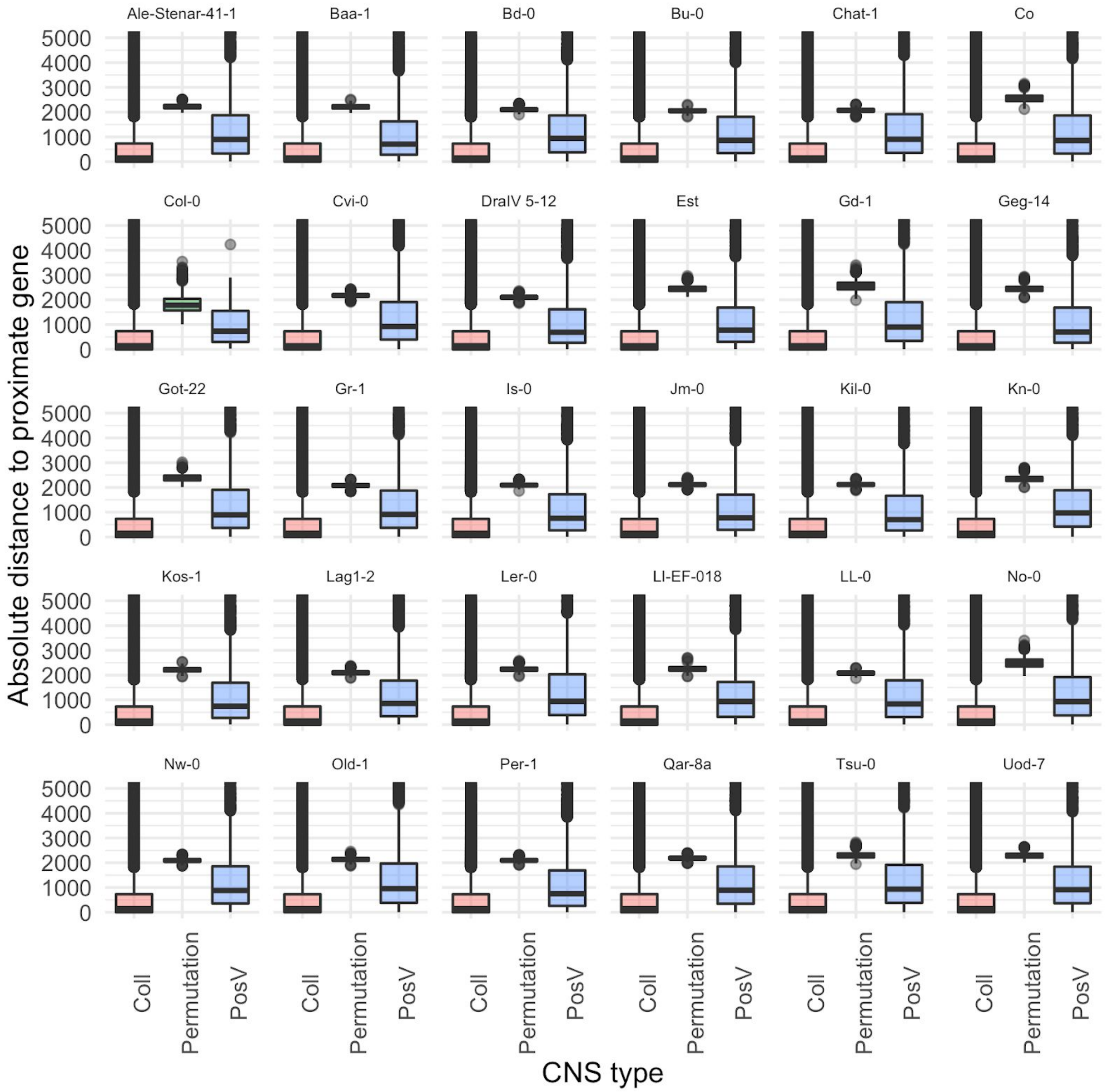

Figure S13:

Figure S13 shows the distribution of (A/B) PiN, (C/D) PiS, and (E/F) PiN/PiS across three classes of genes. The three classes are defined based on the number of CNS associated with them relative to the reference genotype Col-0. The “gain” class genes are orthologous genes in accessions that have more CNS associated with them relative to Col-0. “loss” class genes have less CNS associated with them relative to the reference and “same” genes have the exact same amount. Panels A,C, and E display data for every gene with an ortholog in all thirty accessions (n=20,096). Panels B,D, and F filter these orthologs to cases where there are at least two accessions in each gene class for a given ortholog.

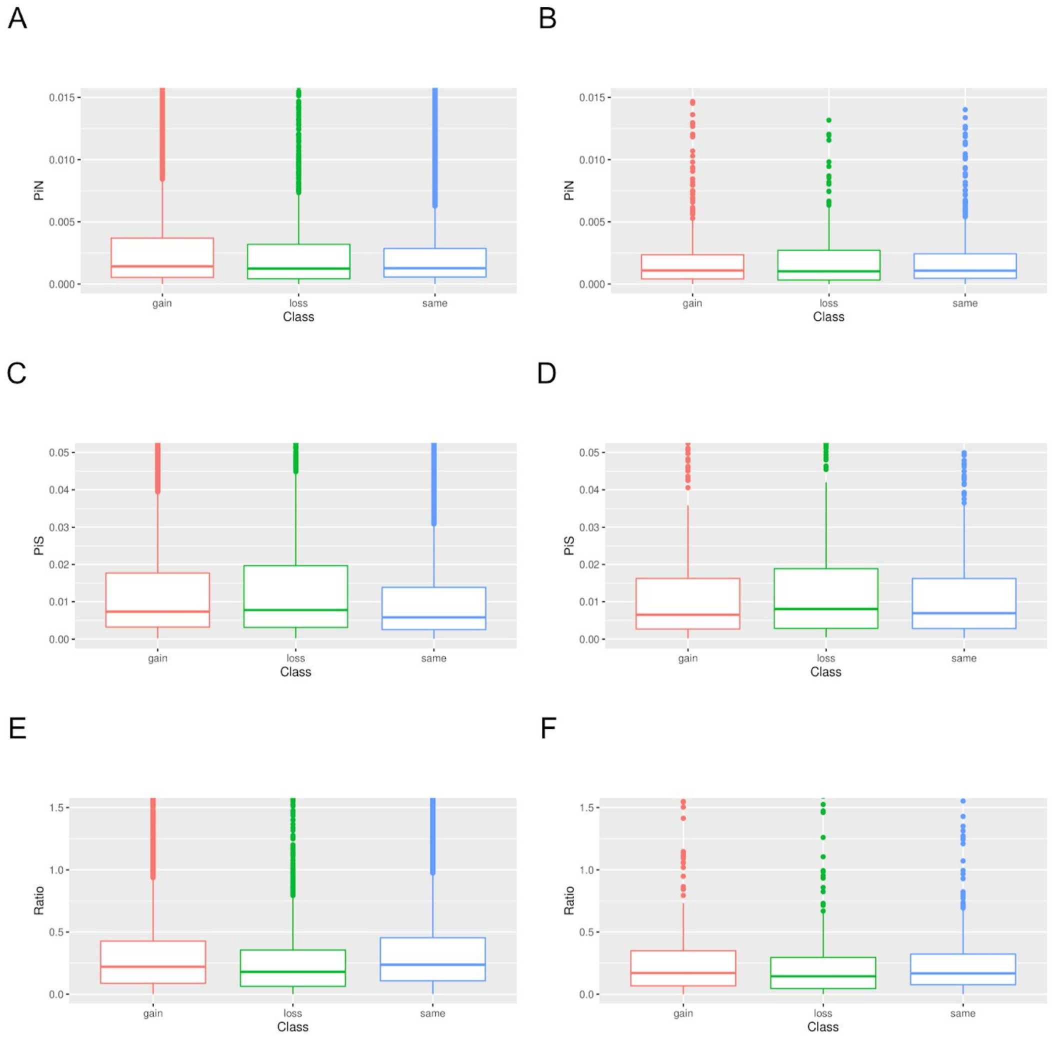

Figure S14:

Figure S14 plots the distance from each CNS separately for all thirty genomes to the proximate repeat annotated by MAKER2 (Holt and Yandell 2011). Collinear CNS and PosV CNS are plotted separately. The locations of PosV CNS have been permuted across the genome 100 separate times (PosV_permutation).

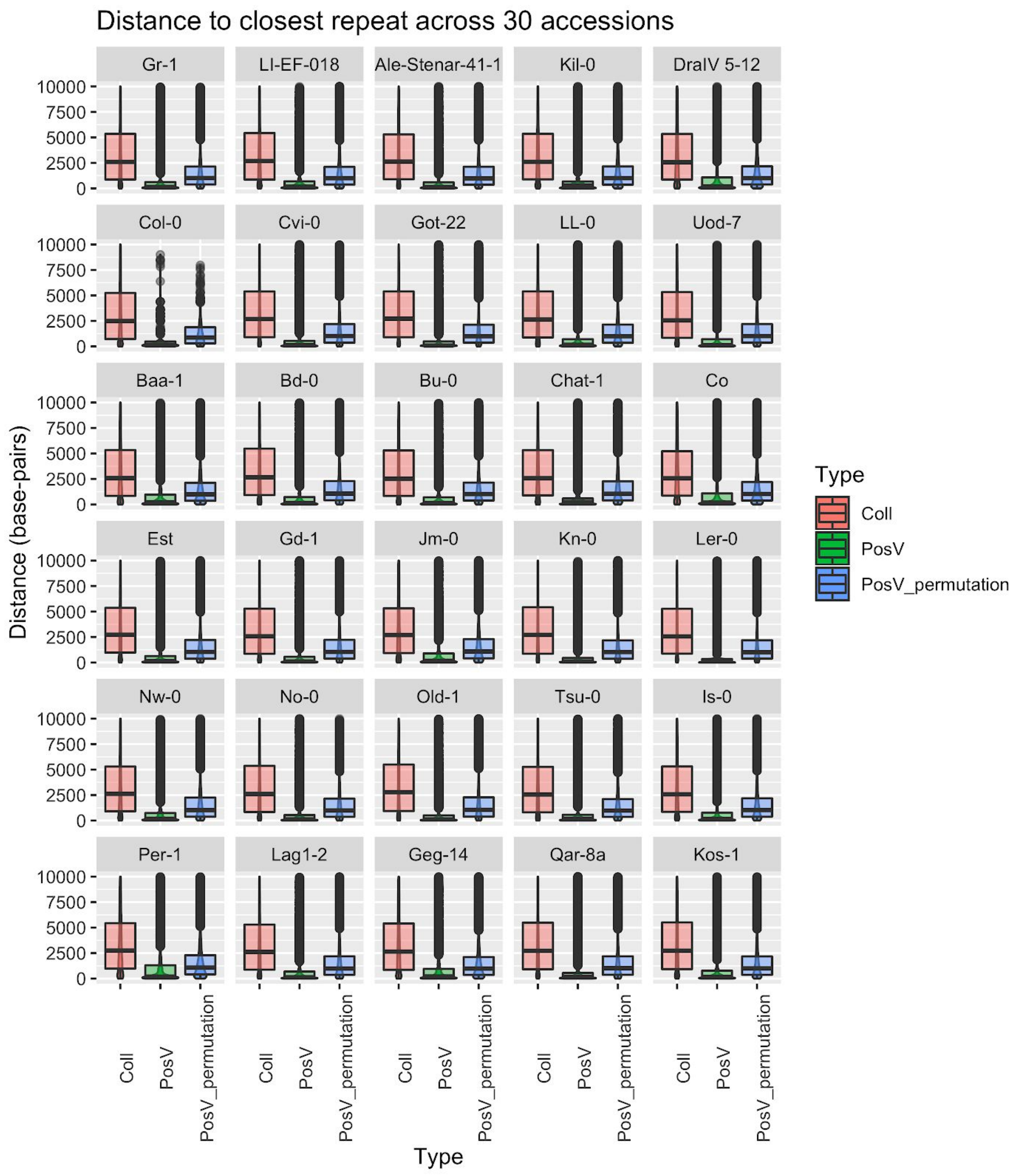

Figure S15:

Figure S15 compares the distribution of CNS counts across all genes in this study between different duplication classifications. This compares the distributions only considering genes with at least one CNS associated with them. The data displayed here is discrete. Therefore a smoothing factor was applied for clarity. The boxplot overlain on the distribution however, aligns on integers representing the 25th,50th, and 75th percentiles. Whiskers extend 1.5x the interquartile range (Q3 - Q1). Duplicate classifications were taken from (Wang et al. 2013).

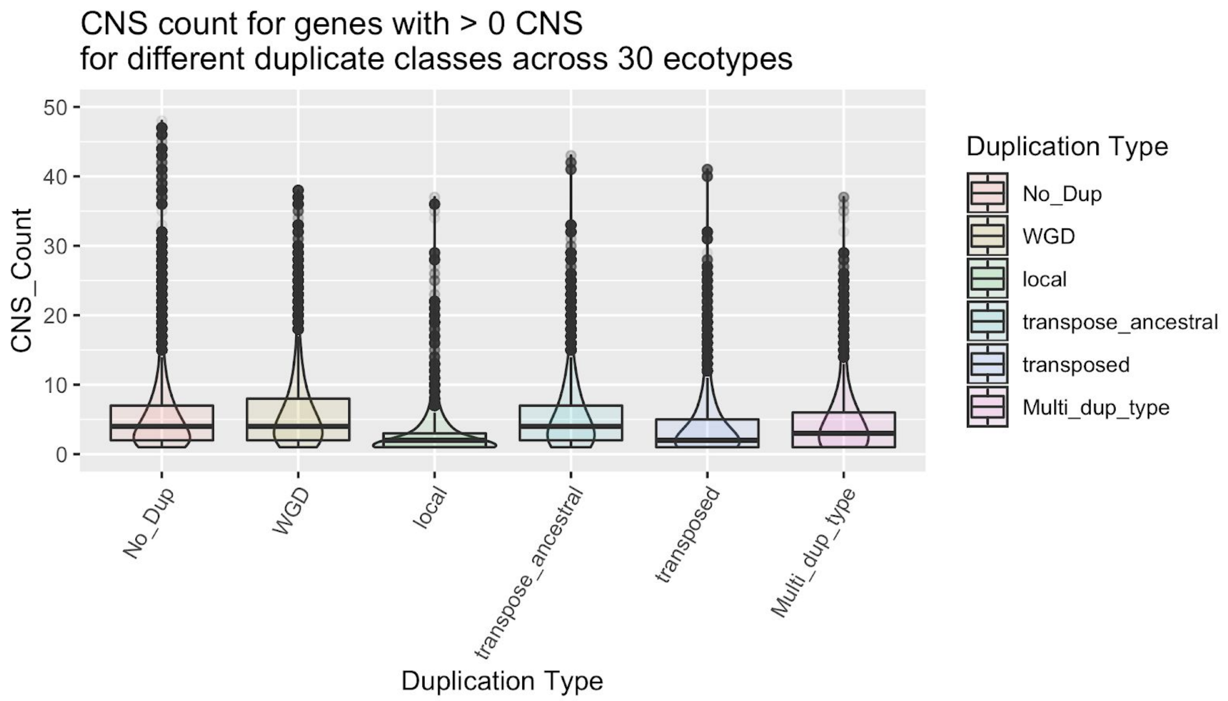

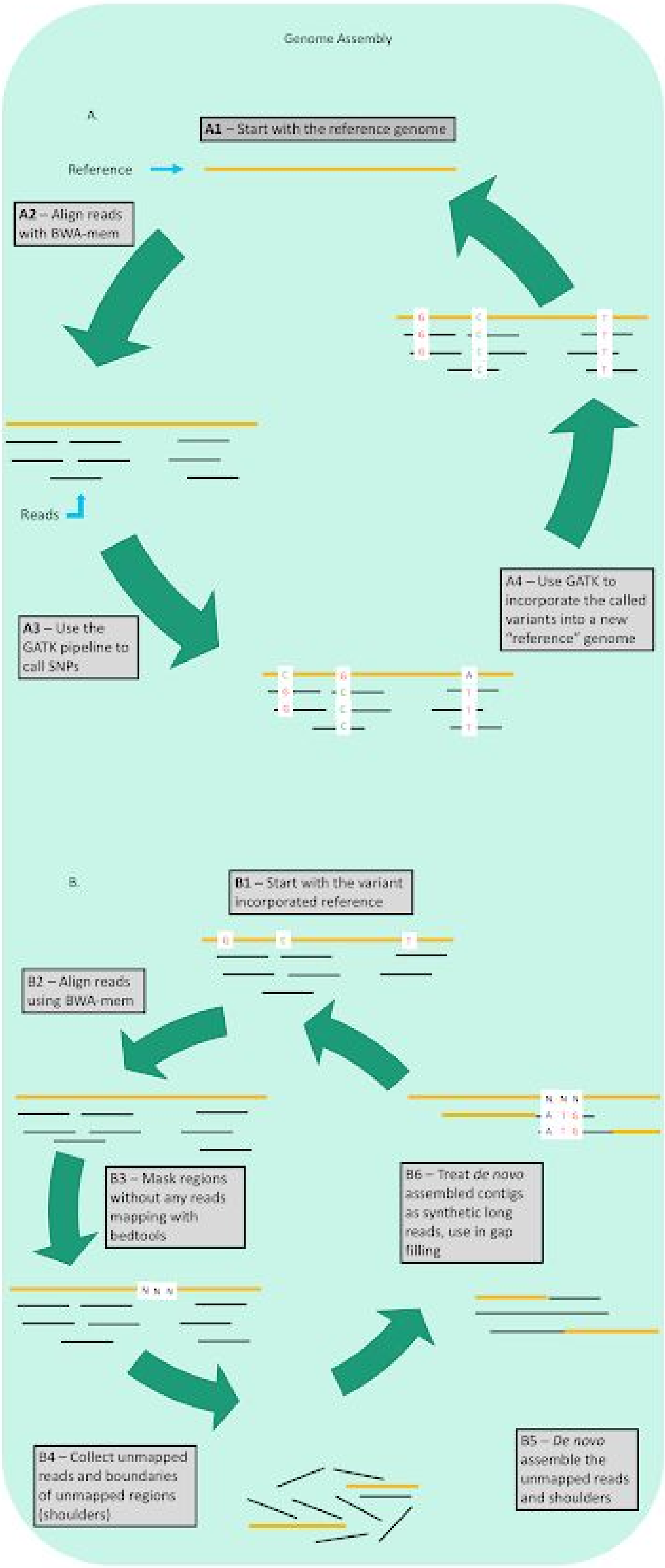

Figure S16:

Figure S16 provides a graphic of the hybrid reference-guided de novo assembly pipeline used in this study.

1001 Genomes Consortium. 2016. 1,135 Genomes Reveal the Global Pattern of Polymorphism in Arabidopsis thaliana. *Cell* 166:481–491.

Conway JR, Lex A, Gehlenborg N. 2017. UpSetR: an R package for the visualization of intersecting sets and their properties. *Bioinformatics* 33:2938–2940.

Emms D. M. KS. 2018. OrthoFinder2: fast and accurate phylogenomic orthology analysis from gene sequences. *bioRxiv* [Internet]. Available from: http://dx.doi.org/10.1101/466201

Holt C, Yandell M. 2011. MAKER2: an annotation pipeline and genome-database management tool for second-generation genome projects. *BMC Bioinformatics* 12:491.

Taiyun Wei And. 2017. R package "corrplot": Visualization of a Correlation Matrix (Version 0.84). Available from: http://dx.doi.org/https://github.com/taiyun/corrplot

Wang Y, Tan X, Paterson AH. 2013. Different patterns of gene structure divergence following gene duplication in Arabidopsis. *BMC Genomics* 14:652.
