# Supplementary material for: Evolution of Conserved Noncoding Sequences in Arabidopsis thaliana
Source: Mol Biol Evol. 2021 Feb 10;38(7):2692–703. doi: 10.1093/molbev/msab042 (PMC8233505; doi:10.1093/molbev/msab042)
Supplement: msab042_Supplementary_Data [file msab042_supplementary_data.zip › Yocca_CNS_Supp_Meth_21_01_27.pdf]

Title: Evolution of conserved noncoding sequences in *Arabidopsis thaliana*
Alan E. Yocca[1,2], Zefu Lu[3], Robert J. Schmitz[3], Michael Freeling[4], Patrick P. Edger[2,5]

1. Department of Plant Biology, Michigan State University, 612 Wilson Rd. East Lansing MI 48823
2. Department of Horticulture, Michigan State University, 1066 Bogue St. East Lansing, MI 48824
3. Department of Genetics, University of Georgia, 120 Green Street, Athens, GA 30602-7223
4. Department of Plant and Microbial Biology, University of California, 111 Koshland Hall, Berkeley, CA 94720
5. Ecology, Evolutionary Biology and Behavior, Michigan State University, East Lansing, MI, USA 48824

Selection of accessions

Accessions were selected to capture great genetic variability in the *A. thaliana* species. Therefore, at least one accession was selected from each of the nine admixture groups defined by the 1001 Genomes Consortium (1001 Genomes Consortium 2016). Seventeen accessions were selected based on availability of high read depth genomic data. Eighteen accessions had ATAC-sequencing data available (Table S2).

CNS length distribution

The length of each CNS was calculated with a custom perl script as shown below.

```
``` $ ./fasta_length_dist_vect.pl -f <input fasta> -o <output> ```
```

The resulting list of lengths was visualized using ggplot2 (Wilkinson 2011) in R v3.5.0 (R Core Team 2018) using the geom_density() function as shown in athal_cns_line_graphs.Rmd.
```

Genome Assembly

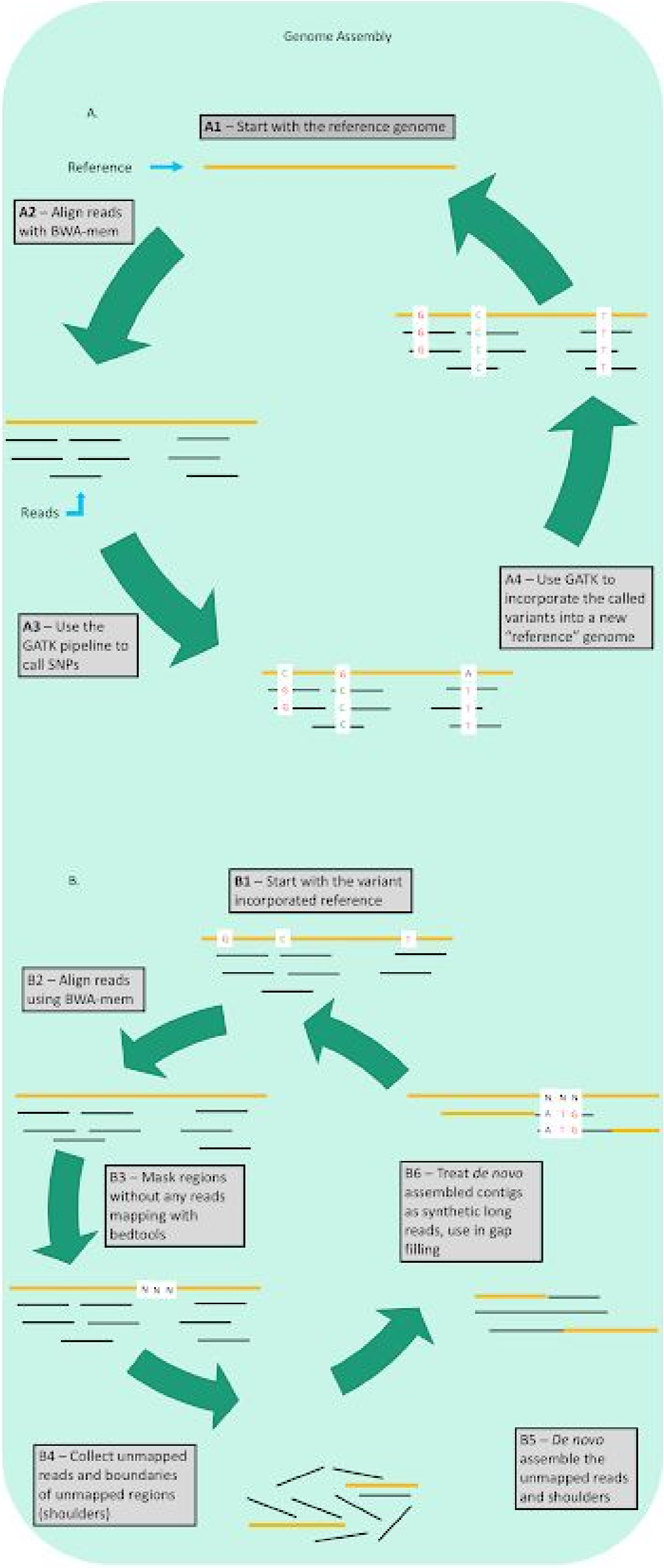

Figure S19: Figure S19 provides a graphic of the hybrid reference-guided *de novo* assembly pipeline used in this study.

A hybrid reference guided and *de novo* assembly approach was taken to assemble the genomes of 30 (29 wild accessions, plus the reference Col-0) separate *Arabidopsis thaliana* accessions. 150 base-pair paired-end reads were retrieved from the NCBI SRA (Table S2). Indexes were created for the genomes to which reads were mapped using SAMTools/1.17 (Li et al. 2009) and BWA/0.7.17 (Li and Durbin 2009) using the following example commands.

``` $ samtools faidx ${CONSENSUS_PATH} ```
``` $ bwa index -p ${BWA_INDEX} ${CONSENSUS_PATH} ```

The reads were mapped to the TAIR10 reference genome using BWA-MEM default parameters and an arbitrary string as a read tag so tools used in later analyses would not raise exceptions.

```
$ bwa mem -R'@RG\tID:group1\tSM:sample1\tPL:illumina\tLB:lib1\tPU:unit1' \
        -t 39 \
        ${BWA_INDEX} \
        ${READ_DIR}/${LINE}_1_paired.fastq \
        ${READ_DIR}/${LINE}_2_paired.fastq \
        > 06_SAM/${TAG}.sam
```

The resulting SAM files were converted to sorted BAM files using Picard Tools v2.18.1 (Anon).

```
$ java -jar $EBROOTPICARD/picard.jar \
        SortSam \
        INPUT=06_SAM/${TAG}.sam \
        OUTPUT=07_BAM/${TAG}.bam \
        SORT_ORDER=coordinate
```

The resulting BAM file was indexed using SAMTools. All indexing of BAM files was performed using the following template command.

```
$ samtools index ${TAG}.bam
```

PCR optical duplicates were marked using Picard Tools. The output BAM file was indexed using SAMTools.

```
$ java -jar $EBROOTPICARD/picard.jar \
        MarkDuplicates \
        M=07_BAM/${TAG}_dup_stats.txt \
        I=07_BAM/${TAG}.bam \
        REMOVE_DUPLICATES=true \
        O=07_BAM/${TAG}_markdup.bam
```

Single Nucleotide Polymorphisms (SNPs) were called using GATK (McKenna et al. 2010). First, insertions and deletions were locally realigned using GATK IndelRealigner. First, realignment target regions were identified.

```
$ java -jar $EBROOTGATK/GenomeAnalysisTK.jar \
        -T RealignerTargetCreator \
        -R ${CONSENSUS_PATH} \
        -I 07_BAM/${TAG}_markdup.bam \
        -o 07_BAM/${TAG}.intervals
```

Then, realignment was performed on those regions. The realigned BAM file was indexed with SAMTools.

```
$ java -jar $EBROOTGATK/GenomeAnalysisTK.jar \
        -T IndelRealigner \
        -R ${CONSENSUS_PATH} \
        -I 07_BAM/${TAG}_markdup.bam \
        -targetIntervals 07_BAM/${TAG}.intervals \
        -o 07_BAM/${TAG}_realign.bam
```

SNPs were identified using GATK UnifiedGenotyper.

```
$ java -Xmx10g -Djava.io.tmpdir=./${LINE}_tmp \
        -jar $EBROOTGATK/GenomeAnalysisTK.jar \
        -R ${CONSENSUS_PATH} \
        -T UnifiedGenotyper \
        -I 07_BAM/${TAG}_realign.bam \
        --out 12_VCF/${TAG}.vcf \
        --num_threads 39 \
        --genotype_likelihoods_model BOTH \
        --sample_ploidy 2 \
        --defaultBaseQualities 30
```

The resulting VCF file was filtered using VCFUtils.pl (lh).

```
$ ${SCRIPT_DIR}/vcfutils.pl \
```

```
        varFilter 12_VCF/${TAG}.vcf \
        > 12_VCF/${TAG}_vf.vcf
```

A consensus sequence was called by incorporating the high confidence SNPs into the reference sequence using GATK FastaAlternateReferenceMaker.

```
$ java -jar $EBROOTGATK/GenomeAnalysisTK.jar \
        -T FastaAlternateReferenceMaker \
        -R ${CONSENSUS_PATH} \
        -o 08_consensus/${TAG}.fasta \
        -V 12_VCF/${TAG}_vf.vcf
```

The resulting consensus sequence was used as the reference sequence for the next round of iterative read mapping. This iterative read mapping and consensus calling method was performed three times, as the number of SNPs between the reads and the consensus after three rounds did not decrease significantly. This completed the reference guided component of the genome assembly

The third consensus genome for each accession was used for further assembly. Reads from each accession were mapped to their respective consensus sequence as described above. Read depth coverage was calculated across the genome to identify regions in the consensus sequence where no reads were mapped.

```
$ bedtools genomecov -bga \
      -ibam 07_BAM/${TAG_LAST}.bam \
      > 13_BED/${TAG_LAST}_all.bed

$ grep -w 0$ 13_BED/${TAG_LAST}_all.bed > 13_BED/${TAG_LAST}_empty.bed
```

Areas in the consensus sequence where no reads were mapped were hard masked using BEDTools/2.27.1 (Quinlan and Hall 2010).

```
$ bedtools maskfasta -fi ${CONSENSUS_PATH} \
      -bed 13_BED/${TAG_LAST}_empty.bed \
      -fo ${CONSENSUS_PATH_BASE}_masked.fasta
```

Unmapped reads were collected using a combination of SAMTools and SeqTK (lh).

```
$ samtools view -S -f4 07_BAM/${TAG_LAST}.bam \
      > 09_UNMAPPED/${TAG_LAST}_um.sam
$ cut -f1 09_UNMAPPED/${TAG_LAST}_um.sam \
      | sort \
      | uniq > 09_UNMAPPED/${TAG_LAST}_um_ids.lst
$ seqtk subseq ${READ_DIR}/${LINE}_1_paired.fastq \
      09_UNMAPPED/${TAG_LAST}_um_ids.lst \
      > 09_UNMAPPED/${TAG_DIR}/${TAG_DOT}.1.um.fastq
$ seqtk subseq ${READ_DIR}/${LINE}_2_paired.fastq \
      09_UNMAPPED/${TAG_LAST}_um_ids.lst \
      > 09_UNMAPPED/${TAG_DIR}/${TAG_DOT}.2.um.fastq
```

200 base-pairs of unmasked sequences flanking masked regions were also extracted using two custom perl scripts `split_fasta_by_Ns.pl` and `fasta_shoulder.pl`.

```
$ ./split_fasta_by_Ns.pl \
      -f ${CONSENSUS_PATH_BASE}_one_line.fasta \
      -o ${CONSENSUS_PATH_BASE}_split.fasta
$ ./fasta_shoulder.pl \
      -f ${CONSENSUS_PATH_BASE}_split.fasta \
      -o ${CONSENSUS_PATH_BASE}_split_shoulder.fasta \
      --min 50 --length 200
```

These sequences were added to the unmapped read files as synthetic short reads. The unmapped sequences and shoulder sequences were fed into MaSuRCA-3.2.6_6.2 (Zimin et al. 2013) for *de novo* assembly. The resulting contigs likely contained sequences that overlapped the unmasked shoulders and masked regions. These were treated as “synthetic long reads”.

The masked consensus sequence was used as a reference for gap filling with the “synthetic long reads” using PBJelly. Six rounds of gap filling were performed, where the previous round of gap filling was used as the reference for the next round. After six rounds, PBJelly was no longer able to fill any more gaps for most accessions. To keep assemblies consistent across accessions, six rounds of gap filling were performed in each accession. The shell script used to perform the iterative gap filling is `viigf_pbj.sbatch`.

We generated an independent assembly of Col-0 to serve as a control for false positive PAV CNS. For newly positioned CNS, PosV, we arbitrarily defined Col-0 positions to be the reference. When a non-reference accession has a CNS at a position not in Col-0, we label that CNS as PosV. The independent Col-0 assembly (Col-0’) was analyzed for variation using the same methods as the other accessions. As Col-0’ and the reference Col-0 genome should have

identical CNS structure, few differences are expected due to methodological differences in assembly and annotation pipelines. Of the 62,916 CNS analyzed, there were only 26 found absent in Col-0' (PAV), compared to an average of 163 displaying PAV in the other accessions examined. More strikingly, only 31 CNS exhibited PosV in Col-0', compared to an average of 910 (~1.45% total CNS) exhibiting PosV per accession. Therefore, our assembly method identified novel PosV and PAV events which are unlikely caused simply by false positive identification attributable to poor or biased assembly quality.

CNS annotation

CNS were annotated in each genome with the BLAST (Altschul et al. 1997) program combined with stringent filtering. CNS themselves were filtered for only those associated with a gene in the TAIR10 annotation. Additionally, CNS showing evidence of encoding any sort of non coding RNA were removed. We did not include non coding RNA loci, as these constitute a minority of identified CNS and likely obey separate functional constraints than the majority of CNS since RNA loci encode for a functional transcribed product. To increase the confidence in CNS annotations, all CNS below 15 base-pairs in length were dropped. After querying each accession's genome with the final set of 62,916 CNS, the resulting hits were filtered to remove any match with a bit score lower than 28.2. Specifically, this corresponds to an exact 15 base-pair match. Hits were also dropped if they covered < 60% of the length of the CNS considered in that hit. Hits on separate chromosomes were also removed.

After stringent filtering of BLAST outputs, any CNS without a hit was considered to exhibit PAV in that accession.

These steps were also followed for the reference genome to allow for accurate annotation of the CNS position in the reference. This annotation was used in comparison with that of each accession to determine CNS which exist outside of syntenic CNS blocks with a block size of 5. This was determined using the MCScanX program (Wang et al. 2012). The script used for CNS annotation in an accession is at_cns01_comp_pipe_cmd.sh. As collinearity was the determining factor in PosV CNS annotation, no minimum distance was required for the classification of a CNS as exhibiting PosV.

Association of CNS with genes

CNS were associated with their proximate gene based on distance regardless of strand. This was performed using BEDTools/2.27.1 "closest" function. The inputs were CNS annotations in BED format and gene annotations in BED format. CNS associations were done separately for collinear CNS and PosV CNS, however this would not affect the results. Keeping them in separate files aided in downstream analysis. Below is an example command used to associate all CNS in the "CNS.bed" file with their proximate gene in the "gene.bed" file:

```
bedtools closest -d -t all -a CNS.bed -b gene.bed > CNS_proximate_gene_closest.txt
```

```

The -t flag reports all in case of ties. Therefore, the same CNS can be counted for two separate genes. We found this to be the case in ~10 CNS per accessions out of a total >64,000 therefore representing a negligible effect. The output of the above command was used to both count the number of CNS associated with each gene, and to calculate the distribution of different classes of CNS around genes.

Importantly, we kept the same gene assignments as discovered in Col-0 for collinear CNS. Therefore, CNS-gene association differences in an accession from Col-0 reflect loss and transposition events only.

## Number of CNS associated with each gene:

The output from the ``` bedtools closest``` command was used to calculate the number of CNS associated with each gene using the custom script gene_cns_count.pl. Briefly, this script reads in a file listing CNS annotations and their proximate gene annotations. For each gene, it counts the number of CNS for which it is the proximate gene. Below is an example command used to count the number of CNS associated with each gene in a given accession:

```
$ ./gene_cns_count.pl --closest_bed ${bedtools_closest_output}.bed \
      --fasta ${accession}.cds --output ${accession}_gene_cns_count.txt \
      --posv_only --spec_file ${accession}_posv_list.txt --spec_tag PosV
```

## ATAC peak calling

ATAC peaks were identified using MACS2 (Zhang et al. 2008). The script to execute the command below is macs2.sh. Peaks with a q-value of 0.1 were used after manually inspecting various peak calling threshold calls against mapped reads. Visual inspection of ATAC-sequencing data led to the removal of three accessions (Nw-0, Old-1, and No-0; Table S1) due to visual inconsistencies between peak calls and sequencing coverage. We still provide files for these accessions, however they were not used to calculate summary statistics. Example visualizations for each accession are included in the dryad submission.

```
$ macs2 callpeak \
-t ${BAM} -g 1.35e8 --keep-dup all \
-n ${OUTPUT} \
-q ${QVALUE}
```
```

Accessible region overlap

The number of CNS overlapping ATAC peaks was performed using BEDTools v2.27.1 (Quinlan 2014). We specify at least half the length of a CNS must overlap with an ATAC peak to be considered overlapping.

```
$ bedtools intersect -a ${CNS}.bed -b ${ATAC}.peaks.narrowPeak -f 0.5 >
${CNS}_overlap_atac.bed
```

Accessible region overlap enrichment

To test for the enrichment of accessible regions overlapping CNS, permutations were performed. By shuffling CNS annotations randomly across the genome, we can generate a distribution of CNS annotations overlapping accessible regions based on chance. Random shuffling of CNS annotations 10,000 times for each accession generated a distribution of the expected number of CNS annotations overlapping accessible regions. Therefore, the true observed overlap between CNS annotations and accessible regions can be compared to this distribution to identify the percentile in the random distribution at which our observed values lie. A percentile value of 1 indicates the observed overlap between CNS annotations and accessible regions exceeds any random overlap across 10,000 permutations. Additionally, we compare the observed overlap of CNS annotations and accessible regions to the mean of the 10,000 permutations. Dividing these values gives us the fold-enrichment over random expectation.

Col-0 Ortholog identification

Syntenic orthologs were identified using JCVI Utilites Library (Tang et al. 2017) between each accession and the reference accession Col-0. The cds sequence of each pair was aligned using MUSCLE v3.8.31 (Edgar 2004).

Subsampling to estimate population level variation captured

To estimate the amount of CNS variation events in the population, CNS variation (PAV and PosV) events were randomly subsampled. For each number of accessions, from 1 to 30, a random subset of taxa was taken 1,000 times. If there were less than 1,000 combinations for a given number of accessions, all possible combinations were taken. For a given random sample, the PAV and PosV CNS were concatenated separately. The number of unique PAV or PosV CNS was calculated from the concatenated list and recorded. The graph generated displays the average unique CNS count across the 1,000 random samples at each number of accessions.

The same procedure was followed for gene variation. The code used to produce these plots are in the R-markdown file athal_cns_line_graphs.Rmd.

We also estimated the expected number of unique CNS PAV and PosV events using the same permutation, however for each permutation, selecting a random set of CNS of similar size.

Determining bias in PosV location

To determine if PosV CNS were preferentially found near genes, we performed a permutation test similar to that performed for calculating the enrichment of CNS overlap with ATAC peaks. We shuffled the PosV annotations across each accession's genome and calculated the distribution of distances from the shuffled CNS to the proximate gene. We compared these distributions to the observed distribution of PosV CNS distances to their proximate gene. This revealed a strong preference for PosV CNS to be closer to genes than expected by chance.

PCA

Principal Component Analysis was performed with the R prcomp() function. We decided not to scale variance, as CNS variability is not normally distributed. The code used for this analysis can be found in the file athal_pca.Rmd. Bioclimatic variables were obtained using the code found in the accompanying file athal_bioclim.Rmd. PCA clustering was also performed on SNP data obtained from the 1001 Genomes Consortium (1001 Genomes Consortium 2016).

RNA-Sequencing

RNA sequencing data was taken for five accessions (Kn-0,Tsu-0,Ler-0,No-0,Col-0), from GSE30814 (Gan et al. 2011). Reads were mapped to their respective assembly using HISAT2/2.1.0 on default parameters (Kim et al. 2015). The resulting SAM file was converted to a BAM file using Picard Tools v2.18.1 as described above. The BAM file was converted to a count matrix using StringTie/1.3.5 along with the accompanying script prepDE.py with minor modifications to handle variable gene names (Pertea et al. 2015). The expression matrices were used to identify differentially expressed genes for each accession to the reference Col-0 using the R package DESeq2 using code provided in the file athal_cns_rna_seq.Rmd (Love et al. 2014).

Motif Enrichment

Enriched motifs were identified using HOMER (Heinz et al. 2010). Enrichment was performed for each accession separately as well as all accessions combined. For each set of accessions, enrichment was performed on PAV CNS and PosV CNS separately. The background set of sequences was set to a set of random sequences given the same composition of the query sequence using the scrambleFasta.pl script provided with HOMER. An example command is shown:

```
scrambleFasta.pl ${FASTA} \
> ${SCRAMBLE_DIR}/${fasta_base}_scramble.fasta
```

Motif enrichment was performed using the following command:

```
findMotifs.pl \
${FASTA} \
fasta \
${OUTPUT} \
-fasta ${SCRAMBLE_DIR}/${fasta_base}_scramble.fasta \
-mset plants \
-p 20
```

Calculation of random occurrences of Kmers

The probability of a random Kmer in a given string was calculated using the following formula in R.

```
Pr <- function(N, A, k, t) {
        return(choose(N - t*(k - 1), t)/A^(t*k))
}
Pr(135000000, 4, 15, 1)
```

“N” represents the size of the string. “A” represents the number of possible characters (four in our case for each of the standard nucleotide bases). “k” represents the size of the Kmer. “t” is the number of occurrences you are testing for (one in our case). This function was taken from https://github.com/wikiselev/bioinformatics-algorithms/wiki/Kmer-expected-number-of-occurrences-in-a-DNA-string.

This is a conservative calculation, as it does not include any sequence composition bias, nor does it account for the genome occurring in five separate chromosomes.

Gene annotation

Gene prediction was performed using MAKER version 2.31.9 (Holt and Yandell 2011) under the parameters shown in the example control file (maker_opts.ctl).

Repeat analysis

Repeat annotations were collected from the output of MAKER2 (Holt and Yandell 2011). The distance for each CNS to the closest repeat annotation was calculated using BEDTools v2.27.1 (Quinlan 2014):

```
bedtools closest -d -t all -nonamecheck -a ${cns}.bed -b ${repeat}.bed >
${overlap}.bed
```

We generated an expected distribution by permuting the location of PosV CNS ten times for each accession. The aggregate of all these permutations is plotted in Figure S16 and S17.

Cruciferous gene identification

We were interested in the rate of gene PAV compared to the rate of CNS PAV. As our set of query CNS exists across a set of nine cruciferous taxa, obtaining a comparable rate of gene PAV must only include genes identifiable in each of these given taxa. Orthofinder2 was used to identify these genes. Briefly, peptide files for each species used in Haudry et al 2013 were collected from the following sources:

**Species**	**Source**
Arabidopsis thaliana	https://www.arabidopsis.org/
Arabidopsis lyrata	ftp://ftp.ensemblgenomes.org/pub/plants/release-44/fasta/arabidopsis_lyrata/pep/Arabidopsis_lyrata.v.1.0.pep.all.fa.gz
Capsella rubella	ftp://ftp.uniprot.org/pub/databases/uniprot/current_release/knowledgebase/reference_proteomes/Eukaryota/UP000029121_81985.fasta.gz
Eutrema salsugineum	ftp://ftp.uniprot.org/pub/databases/uniprot/current_release/knowledgebase/reference_proteomes/Eukaryota/UP000030689_72664.fasta.gz
Schrenkiella parvula	http://biosci-ryo.lsu.edu/files/Sparvula_genome_annotation_v2.0.protein.fa

Brassica rapa	ftp://ftp.ensemblgenomes.org/pub/plants/release-44/fasta/brassica_rapa/pep/Brassica_rapa.Brapa_1.0.pep.all.fa.gz
Aethionema arabicum	https://genomevolution.org/coge/: Aethionema arabicum (formerly known as 'Dick') version 2.5 id23428
Sisymbrium irio	https://genomevolution.org/coge/: Sisymbrium irio (v0.2 id19579)
Leavenworthia alabamica	https://genomevolution.org/coge/: Leavenworthia alabamica (formerly known as 'Tom') v0.2 id19577

Table S4: Table S4 lists the species and source of each peptide file used to identify a set of genes present in each species used to identify CNS by Haudry et al 2013.

Orthofinder2 was run with the following command with the ${SEARCH} variable set to the diamond algorithm.

```
./orthofinder -f ${DIR} -S ${SEARCH} -t ${THREADS}
```

Long-read genome comparison

We were interested whether publicly available long read genomes would return similar results. Therefore, we downloaded long-read genome assemblies for seven *A. thaliana* ecotypes and processed them with the same pipeline run on our genome assemblies to identify PAV and PosV CNS (Wen-Biao Jiao & 2020). Two of these seven accessions were identical to those for which we generated assemblies. Results are shown in Table S5 and Table S6. PAV and PosV CNS between our assemblies and the long read assemblies overlap greatly. The five long-read assemblies representing accessions for which we did not generate assemblies contained PAV and PosV CNS estimates in line with ours (Table S2: Yocca, et. al. minimum PAV CNS = 128, maximum PAV CNS = 333, minimum PosV CNS = 744, maximum PosV CNS = 1321).

Accession	Jiao, Schneeberger PAV CNS (% shared)	Overlap with Yocca, et. al. assembly (%)	Yocca et. al. PAV CNS (% shared)
Cvi	240 (71.7%)	172	214 (80.4%)

Ler	174 (54.6%)	95	137 (69.3%)
Sha	197	N/A	N/A
C24	187	N/A	N/A
Eri	255	N/A	N/A
Kyo	235	N/A	N/A
An-1	174	N/A	N/A

Table S5: Table S5 displays the counts of PAV CNS identified searching long read genome assemblies from Jiao, Schneeberger 2019. Additionally, if available, we report the number of PAV CNS identified in the long-read genome assembly also found in our genome assembly. N/A indicates we did not generate an assembly for the given accession.

Accession	Jiao, Schneeberger PosV CNS (% shared)	Overlap with Yocca, et. al. assembly (%)	Yocca et. al. PosV CNS (% shared)
Cvi	1200 (92.0%)	1104	1194 (92.5%)
Ler	860 (88.5%)	761	808 (94.2%)
Sha	954	N/A	N/A
C24	1007	N/A	N/A
Eri	886	N/A	N/A
Kyo	908	N/A	N/A
An-1	844	N/A	N/A

Table S6: Table S6 displays the counts of PosV CNS identified searching long read genome assemblies from Jiao, Schneeberger 2019. Additionally, if available, we report the number of PosV CNS identified in the long-read genome assembly also found in our genome assembly. N/A indicates we did not generate an assembly for the given accession.

Independent occurrence of CNS variation

If CNS variation occurred randomly, we expect 4,567 and 21,699 different CNS to be lost and positionally variable respectively in at least a single accession. We arrive at this figure using the Linearity of Expectation which states the expected value of the sum of random variables is equal to the sum of their individual expected values (https://brilliant.org/wiki/linearity-of-expectation/). Therefore, we can separately calculate the probability we observe a CNS display PAV or PosV (separate expectation calculations) in at least one of our twenty-nine wild accessions. Summing this probability across all CNS gives us the expected number of distinct CNS displaying PAV (4,566.33) or PosV (21,698.48) if these draws were random. Code to calculate these expectations can be found in athal_cns_line_graphs.Rmd.

PiN/PiS calculation

PiN and PiS we calculated using the program dNdSpiNpiS_1.0. Individual genes were split according to the number of CNS associated with a gene in a given accession relative to the reference genotype. For example, all accessions for which the gene ATXGNNNNN has less CNS associated with it than the reference genotype were put in the same file and aligned. This resulted in PiN/PiS values across gene alignments for three different classes of genes: Those "losing" a CNS relative to the reference, those "gaining" a CNS relative to the reference, and those with the same amount of associated CNS relative to the reference. This resulted in three different classes for comparison: gain, loss, and same.

Nucleotide Diversity Calculation

Nucleotide diversity was calculated using the dendropy package implemented in python (Sukumaran and Holder 2010). The same alignments used for the PiN/PiS analysis were loaded using the dendropy DnaCharacterMatrix.get() function. The dendropy nucleotide_diversity() function was used to calculate nucleotide diversity values for each of these alignments. Nucleotide diversity values for the different types of alignments (gain, loss, and same as defined above) were compared. The code used in this analysis can be found in the file nucleotide_diversity_calc.py.

Bioclimatic Associations

A background model for relatedness across *Arabidopsis thaliana* lines was generated from genome-wide SNP polymorphism data amongst the 1001 genome project lines (https://1001genomes.org/data/GMI-MPI/releases/v3.1/1001genomes_snp-short-indel_only_ACGTN.vcf.gz). Vcftools was used to parse a subset of markers for those that were biallelic, indel-free, had MAF>0.05, a call rate>=75%, quality>=30 and a depth>=5 (Danecek et al. 2011). TASSEL5 (Bradbury et al. 2007) was then used to convert this subset vcf to hapmap format and

GAPIT (Lipka et al. 2012) generated a 3-axis background PCA of kinship. Separately we recoded presence absence variation and positional variation (1/0 to G/A) and again used GAPIT to generate a PCA of PAV and POSV - primarily focussing on PC1 for both cases as a proxy for PAV/POSV phenotype. We then generated a series of linear regression models with PAV/POSV phenotype as the dependent variable and (i) 3 PCA kinship axes, (ii) 3 PCA kinship axes + Bioclim1, as independent variables. The Pearson correlation coefficients with/without Bioclim1 as a covariate are reported.

1001 Genomes Consortium. 2016. 1,135 Genomes Reveal the Global Pattern of Polymorphism in Arabidopsis thaliana. *Cell* 166:481–491.

Altschul SF, Madden TL, Schäffer AA, Zhang J, Zhang Z, Miller W, Lipman DJ. 1997. Gapped BLAST and PSI-BLAST: a new generation of protein database search programs. *Nucleic Acids Res.* 25:3389–3402.

Anon. Picard Tools - By Broad Institute. Available from: http://broadinstitute.github.io/picard/

Bradbury PJ, Zhang Z, Kroon DE, Casstevens TM, Ramdoss Y, Buckler ES. 2007. TASSEL: software for association mapping of complex traits in diverse samples. *Bioinformatics* 23:2633–2635.

Danecek P, Auton A, Abecasis G, Albers CA, Banks E, DePristo MA, Handsaker RE, Lunter G, Marth GT, Sherry ST, et al. 2011. The variant call format and VCFtools. *Bioinformatics* 27:2156–2158.

Duarte JM, Wall PK, Edger PP, Landherr LL, Ma H, Pires JC, Leebens-Mack J, dePamphilis CW. 2010. Identification of shared single copy nuclear genes in Arabidopsis, Populus, Vitis and Oryza and their phylogenetic utility across various taxonomic levels. *BMC Evol. Biol.* 10:61.

Edgar RC. 2004. MUSCLE: multiple sequence alignment with high accuracy and high throughput. *Nucleic Acids Research* [Internet] 32:1792–1797. Available from: http://dx.doi.org/10.1093/nar/gkh340

Gan X, Stegle O, Behr J, Steffen JG, Drewe P, Hildebrand KL, Lyngsoe R, Schultheiss SJ, Osborne EJ, Sreedharan VT, et al. 2011. Multiple reference genomes and transcriptomes for Arabidopsis thaliana. *Nature* 477:419–423.

Heinz S, Benner C, Spann N, Bertolino E, Lin YC, Laslo P, Cheng JX, Murre C, Singh H, Glass CK. 2010. Simple Combinations of Lineage-Determining Transcription Factors Prime cis-Regulatory Elements Required for Macrophage and B Cell Identities. *Molecular Cell* [Internet] 38:576–589. Available from: http://dx.doi.org/10.1016/j.molcel.2010.05.004

Holt C, Yandell M. 2011. MAKER2: an annotation pipeline and genome-database management tool for second-generation genome projects. *BMC Bioinformatics* 12:491.

Kim D, Langmead B, Salzberg SL. 2015. HISAT: a fast spliced aligner with low memory requirements. *Nat. Methods* 12:357–360.

lh. lh3/samtools. *GitHub* [Internet]. Available from: https://github.com/lh3/samtools

lh. lh3/seqtk. *GitHub* [Internet]. Available from: https://github.com/lh3/seqtk

Li H, Durbin R. 2009. Fast and accurate short read alignment with Burrows-Wheeler transform. *Bioinformatics* 25:1754–1760.

Li H, Handsaker B, Wysoker A, Fennell T, Ruan J, Homer N, Marth G, Abecasis G, Durbin R, 1000 Genome Project Data Processing Subgroup. 2009. The Sequence Alignment/Map

format and SAMtools. *Bioinformatics* [Internet] 25:2078–2079. Available from: http://dx.doi.org/10.1093/bioinformatics/btp352

Lipka AE, Tian F, Wang Q, Peiffer J, Li M, Bradbury PJ, Gore MA, Buckler ES, Zhang Z. 2012. GAPIT: genome association and prediction integrated tool. *Bioinformatics* 28:2397–2399.

Love MI, Huber W, Anders S. 2014. Moderated estimation of fold change and dispersion for RNA-seq data with DESeq2. *Genome Biol.* 15:550.

McKenna A, Hanna M, Banks E, Sivachenko A, Cibulskis K, Kernytsky A, Garimella K, Altshuler D, Gabriel S, Daly M, et al. 2010. The Genome Analysis Toolkit: a MapReduce framework for analyzing next-generation DNA sequencing data. *Genome Res.* 20:1297–1303.

Pertea M, Pertea GM, Antonescu CM, Chang T-C, Mendell JT, Salzberg SL. 2015. StringTie enables improved reconstruction of a transcriptome from RNA-seq reads. *Nat. Biotechnol.* 33:290–295.

Quinlan AR. 2014. BEDTools: The Swiss-Army Tool for Genome Feature Analysis. *Current Protocols in Bioinformatics* [Internet] 47:11.12.1–11.12.34. Available from: http://dx.doi.org/10.1002/0471250953.bi1112s47

Quinlan AR, Hall IM. 2010. BEDTools: a flexible suite of utilities for comparing genomic features. *Bioinformatics* 26:841–842.

R Core Team. 2018. R: A language and environment for statistical computing. *R Foundation for Statistical Computing* [Internet]. Available from: https://www.R-project.org/

Stamatakis A. 2015. Using RAxML to Infer Phylogenies. *Current Protocols in Bioinformatics* [Internet]:6.14.1–6.14.14. Available from: http://dx.doi.org/10.1002/0471250953.bi0614s51

Sukumaran J, Holder MT. 2010. DendroPy: a Python library for phylogenetic computing. *Bioinformatics* 26:1569–1571.

Tang H, Krishnakumar V, Li J, Tiany, MichelMoser, Maria, Yim WC. 2017. tanghaibao/jcvi: JCVI v0.7.5. Available from: https://zenodo.org/record/846919

Wang Y, Tang H, Debarry JD, Tan X, Li J, Wang X, Lee T-H, Jin H, Marler B, Guo H, et al. 2012. MCScanX: a toolkit for detection and evolutionary analysis of gene synteny and collinearity. *Nucleic Acids Res.* 40:e49.

Wen-Biao Jiao &. 2020. Chromosome-level assemblies of multiple Arabidopsis genomes reveal hotspots of rearrangements with altered evolutionary dynamics. *Nat. Commun.* [Internet]. Available from: http://dx.doi.org/ https://doi.org/10.1038/s41467-020-14779-y

Wilkinson L. 2011. ggplot2: Elegant Graphics for Data Analysis by WICKHAM, H. *Biometrics* [Internet] 67:678–679. Available from: http://dx.doi.org/10.1111/j.1541-0420.2011.01616.x

Zhang Y, Liu T, Meyer CA, Eeckhoute J, Johnson DS, Bernstein BE, Nussbaum C, Myers RM, Brown M, Li W, et al. 2008. Model-based Analysis of ChIP-Seq (MACS). *Genome Biology* [Internet] 9:R137. Available from: http://dx.doi.org/10.1186/gb-2008-9-9-r137

Zimin AV, Marçais G, Puiu D, Roberts M, Salzberg SL, Yorke JA. 2013. The MaSuRCA genome assembler. *Bioinformatics* [Internet] 29:2669–2677. Available from: http://dx.doi.org/10.1093/bioinformatics/btt476
